# Trends in Mean Energy and Nutrient Intakes in Japanese Children and Adolescents: The National Health and Nutrition Survey, 1995–2019

**DOI:** 10.3390/nu15153297

**Published:** 2023-07-25

**Authors:** Chisa Shinsugi, Hidemi Takimoto

**Affiliations:** Department of Nutritional Epidemiology and Shokuiku, National Institute of Health and Nutrition, National Institutes of Biomedical Innovation, Health and Nutrition, Osaka 566-0002, Japan

**Keywords:** nutrient intakes, National Health and Nutrition Survey, children, adolescents, Japan

## Abstract

This study aimed to describe the national trends in mean energy and nutrient intakes in Japanese children and adolescents from 1995 to 2019. We used data obtained from the National Health and Nutrition Survey and included 54,871 participants aged 1–19 years. The dietary intake was estimated using a 1-day, semi-weighed, household-based, dietary record. The trends of mean energy and nutrient intakes were analyzed using the Joinpoint Regression Program. A declining trend in the mean energy intake was observed in toddlers aged 1–6 years, school girls aged 7–14 years, and adolescent girls aged 15–19 years, while the mean energy intake from protein, fat, and carbohydrates changed little over time. The mean salt equivalent showed a decreasing trend in all age groups, although the 2019 mean values were above the tentative dietary goal for preventing lifestyle-related diseases, especially in adolescent boys. Declining trends in mean vitamin (vitamin A, vitamin B12, folate, vitamin D, and vitamin K) and mineral (calcium, iron, and copper) intakes were observed, while trends in the mean vitamin B6 and zinc intakes were unchanged since 2001. Continuous monitoring of dietary intake and further research are required to raise awareness of unhealthy diet habits and to improve the food environment for the healthy growth and development of children and adolescents.

## 1. Introduction

Dietary habits tend to change over time. Japanese adults have reported a decrease in total energy intake, energy intake from protein, and sodium intake, and an increase in energy intake from fat [1]. Moreover, higher-income groups have shown a trend toward a lower cereal intake in the past decade [2]. Children and adolescents who primarily share meals with their parents may also experience similar changes in dietary intake, but little is known about trends in nutrient intake in this age group. There is a high prevalence of stunted children younger than 5 years (7.1%, 2010) and a high percentage of underweight adolescents (19.8%, 2015) with increasing trends since the 1980s [3]. Therefore, assessing the intake status of essential nutrients for adequate child growth and development is important to determine countermeasures that will prevent child malnutrition.

In Japan, school lunches have been provided to children aged 6–14 years for approximately 70 years in accordance with the School Lunch Program Act. This act incorporates more than one third of the daily requirement for children with a moderate level of physical activity, as indicated in the Dietary Reference Intakes for Japanese (DRIs) [4,5]. Therefore, the nutrient intake of lunch meals in this age group should be stable. However, whether the daily intake, including other meals (breakfast and dinner), and the daily nutrient intake of preschool children and adolescents who do not receive school lunches, are adequate or insufficient over time remain unclear.

Monitoring the nutrient intake of children and adolescents is critical for assessing whether their nutritional requirements for growth and development are being met. The National Nutrition Survey (NNS), later renamed the National Health and Nutrition Survey (NHNS) in 2003, is an annual nationwide cross-sectional survey that has evaluated nutrient intakes for Japanese aged ≥1 year for longer than 70 years. Therefore, we aimed to describe the national trends in mean energy and nutrient intakes in children and adolescents from 1995 to 2019.

## 2. Materials and Methods

### 2.1. Study Participants

We used data from published survey reports from the NNS (1995–2002) and the NHNS (2003–2019) conducted by the Ministry of Health, Labour and Welfare of Japan [6]. The period of this study (1995–2019) was selected because the assessment of individual intake of nutrients and food groups began in 1995. Before this time, only household-based food consumption was assessed. The 2020 and 2021 NHNSs were canceled owing to the effect of the coronavirus disease 2019 pandemic. Therefore, the 2019 NHNS has the most recent data available. The study participants were selected using a stratified cluster sampling design across all 47 prefectures (Japanese equivalent to province). Residents aged ≥1 year in all households were selected from 300 census enumeration areas, except for the 2004, 2012, and 2016 surveys, which were conducted in 298 (2 areas in Niigata Prefecture were excluded owing to the Mid-Niigata Prefecture Earthquake), 475, and 462 areas, respectively. The method of cluster sampling in 2012 and 2016 differed from that in the other years, and weighing was performed to correct for differences between the number of households in each prefecture. Three prefectures (Iwate, Miyagi, and Fukushima) were excluded from the 2011 survey because of the Great East Japan earthquake, and one prefecture (Kumamoto) was excluded from the 2016 survey owing to the Kumamoto earthquake. The detailed methodology of the NNS and NHNS have been described elsewhere [7,8]. On the basis of official application procedures under Article 33 of the Statistics Act, we obtained approval for data use from the Ministry of Health, Labor, and Welfare. Ethical review and approval were waived for this study because only anonymized data were used.

Of the 1995–2019 survey participants, 54,871 (28,062 boys and 26,809 girls) who were aged 1–19 years were included in this analysis.

### 2.2. Dietary Assessment

Data on dietary intake were collected using a 1-day, semi-weighed, household-based, dietary record on a single day that was neither a Sunday nor a public holiday. The individuals who usually cook for the family recorded the names of food ingredients, weight, and the leftover amount of food for each child in the dietary record. When food weight was missing, trained dieticians estimated the food weight using an official food item booklet with standard portion sizes for frequently consumed dishes. To account for shared dishes within the household, the approximate proportions of each food were assigned to individual household members to estimate the individual food intake. School lunch recipes were collected from the educational board in charge in the case of local public schools and schools serving school lunches in the case of private schools. The individuals who usually cook for the family asked their children about the amount eaten at school lunch and recorded this information. During the survey, trained dietitians visited each household at least once a day to check the dietary record.

The nutrient intake was calculated using the Standard Tables of Food Composition in Japan (4th edition for the 1995–2000 survey; 5th edition for the 2001–2004 survey; 5th revised and enlarged edition for the 2005–2010 survey; 2010 edition for the 2011–2017 survey; 2015 edition for the 2018–2019 survey). Of the 38 energy and nutrient intakes observed in the NHNS [9], the following data on key nutrients for child growth and development based on the DRIs [10] and the American Academy of Pediatrics (AAP) statements [11] were examined in this study: energy, protein, total fat, saturated fat, cholesterol, carbohydrates, total dietary fiber, sodium, salt, energy from carbohydrates, energy from protein, energy from fat, vitamin A (retinol equivalent), thiamine, riboflavin, niacin equivalent, vitamin B6, vitamin B12, folate, pantothenic acid, vitamin C, vitamin E (alpha-tocopherol), vitamin K, potassium, calcium, magnesium, phosphorus, iron, zinc, and copper. Energy and major nutrient intakes have been reported since 1995, while the intake of some detailed nutrients, such as dietary fiber, began to be reported in 2001.

### 2.3. Statistical Analysis

The mean and standard deviation (SD) were calculated by sex, three age categories (1–6, 7–14, and 15–19 years), and the survey year. The trend analyses were performed using Stata Version 16.1 (StataCorp, College Station, TX, USA) and the Joinpoint Regression Program (Joinpoint Regression software, version 4.9.1.0; National Cancer Institute, Rockville, MD, USA) [12]. Joinpoint regression analysis uses statistical criteria to determine the minimum number of linear segments required to describe a trend and perform the annual percentage change (APC) for each segment. The Monte Carlo Permutation method was used to test if a change in the trend was statistically significant. Differences were considered statistically significant at *p* < 0.05.

## 3. Results

The trends in energy and nutrient intakes according to sex and age are shown in Table 1 (young boys aged 1–6 years), Table 2 (young girls aged 1–6 years), Table 3 (school boys aged 7–14 years), Table 4 (school girls aged 7–14 years), Table 5 (adolescent boys aged 15–19 years), and Table 6 (adolescent girls aged 15–19 years). The number of survey participants decreased over the years.

**Figure 1:**
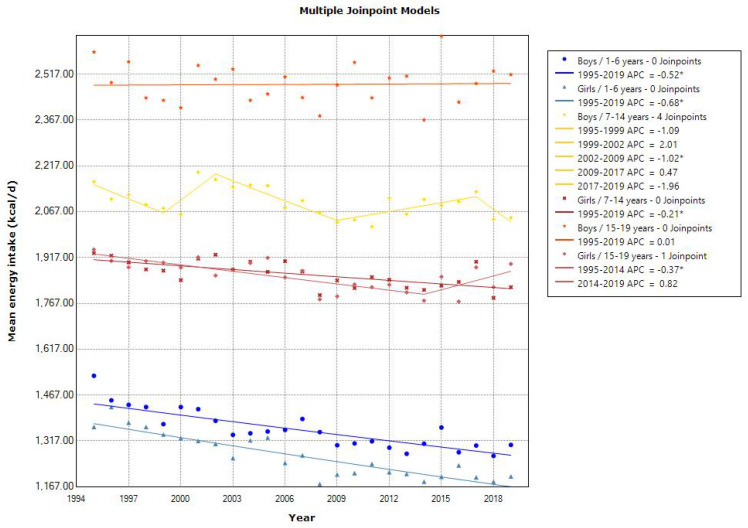
Trends in the proportion of energy intake according to sex and age categories. APC, annual percentage change. * *p* < 0.05.

**Figure 2:**
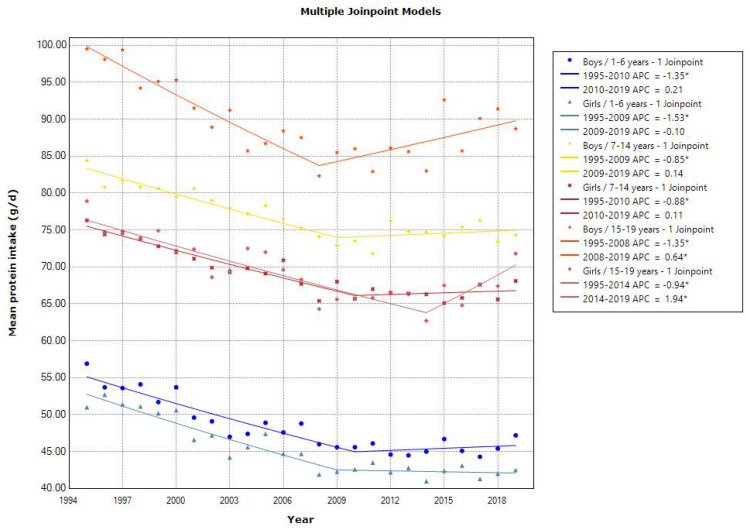
Trends in the proportion of protein intake according to sex and age categories. APC, annual percentage change. * *p* < 0.05.

**Figure 3:**
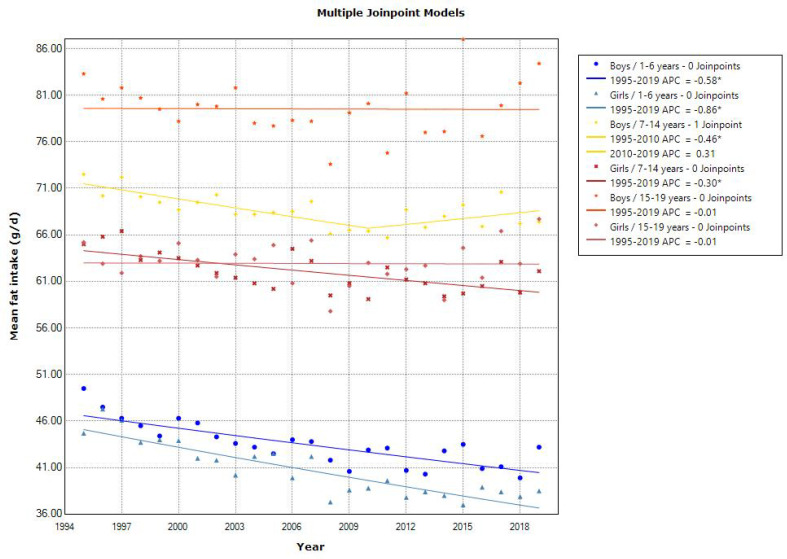
Trends in the proportion of fat intake according to sex and age categories. APC, annual percentage change. * *p* < 0.05.

**Figure 4:**
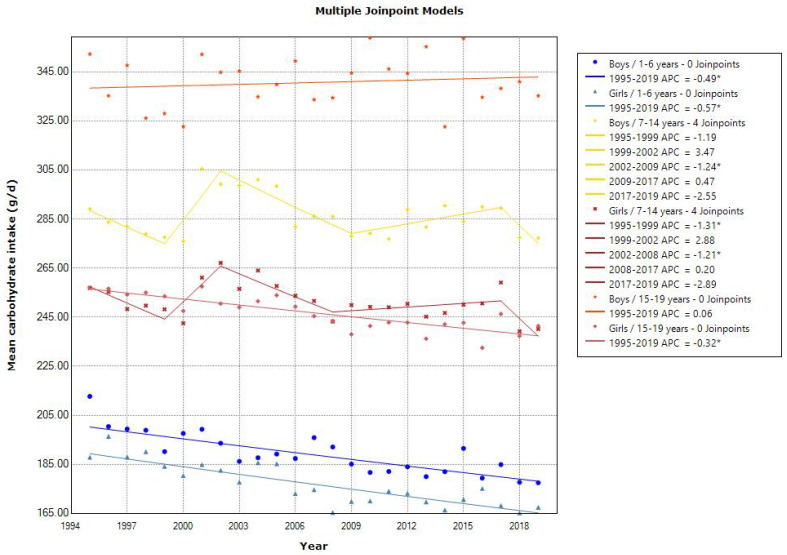
Trends in the proportion of carbohydrate intake according to sex and age categories. APC, annual percentage change. * *p* < 0.05.

Joinpoint regression analyses showed a declining trend in the mean energy intake in toddlers aged 1–6 years (APC of −0.52 for boys and −0.68 for girls, *p* < 0.001), school girls aged 7–14 years (APC of −0.21, *p* < 0.001), and adolescent girls aged 15–19 years (APC of −0.37 [1995–2014]) (Figure 1). The latest mean energy intake in 2019 was almost within the estimated energy requirement in the 2020 DRIs, which was set at 900–1300, 1250–2900, and 1700–3150 kcal/day for 1–5, 6–14, and 15–19 years, respectively [10]. Additionally, the mean energy intake from protein, fat, and carbohydrates was within the tentative dietary goal for preventing lifestyle-related disease (DG) for energy intake from protein (13–20% energy), fat (20–30% energy), and carbohydrates (50–65% energy) in the 2020 DRIs, except for energy intake from fat in school and adolescent girls. Similarly, a decreasing trend in the mean protein intake was observed in toddlers aged 1–6 years (APC of −1.35 for boys [1995–2010] and APC of −1.53 for girls [1995–2009], *p* < 0.001) and school children aged 7–14 years (APC of −0.85 for boys [1995–2009] and −0.88 for girls [1995–2010], *p* < 0.001). However, adolescents aged 15–19 years showed a decreasing and then increasing trend in the mean protein intake (APC of −1.35 [1995–2008] and 0.64 [2008–2019] for boys, and APC of −0.94 [1995–2014] and 1.94 [2014–2019] for girls, *p* < 0.001) (Figure 2). A declining trend in the mean fat intake was observed in toddlers aged 1–6 years (APC of −0.58 for boys and −0.86 for girls, *p* < 0.001) and in school children aged 7–14 years (APC of −0.46 for boys [1995–2010] and −0.30 for girls, *p* < 0.001). However, the mean fat intake was unchanged in adolescents (Figure 3). A declining trend in the mean carbohydrate intake was found in toddlers aged 1–6 years (APC of −0.49 for boys and −0.57 for girls, *p* < 0.001) and in adolescent girls aged 15–19 years (APC of −0.32, *p* < 0.001) (Figure 4).

The intake of total dietary fiber has been reported since 2001. The intakes of saturated fat, sodium, and energy from protein in 2001 and 2004 were not described in the respective survey reports.

**Figure 5:**
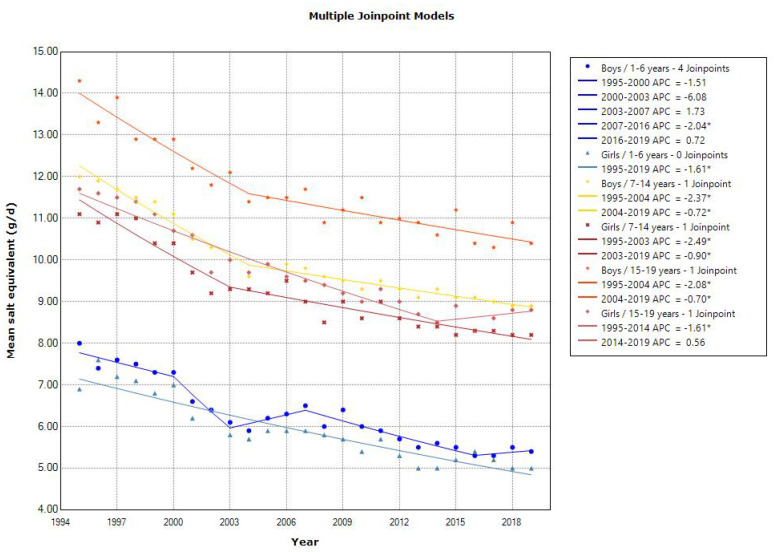
Trends in the proportion of the salt equivalent according to sex and age categories. APC, annual percentage change. * *p* < 0.05.

A decreasing trend in the mean salt equivalent was observed in toddlers aged 1–6 years (APC of −2.04 for boys [2007–2016] and APC of −1.61 for girls), school children aged 7–14 years (APC of −2.37 [1995–2004] and −0.72 [2004–2019] for boys, and APC of −2.49 [1995–2003] and −0.90 [2003–2019] for girls), and adolescents aged 15–19 years (APC of −2.08 [1995–2004] and −0.70 [2004–2019] for boys, and APC of −1.61 [1995–2014] for girls) (Figure 5). The latest mean salt equivalent in 2019 was above the DG in the 2020 DRIs (<3.0–3.5, 4.5–7.0, and 6.5–7.5 g/day for 1–5, 6–14, and 15–19 years, respectively).

**Figure 6:**
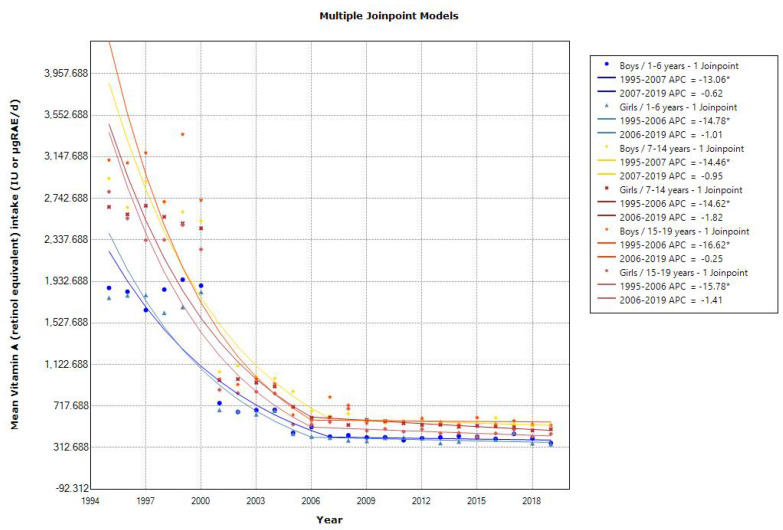
Trends in the proportion of vitamin A intake according to sex and age categories. APC, annual percentage change. * *p* < 0.05. Prior to 2000, International Unit (IU) was used as the unit of vitamin A intake.

**Figure 7:**
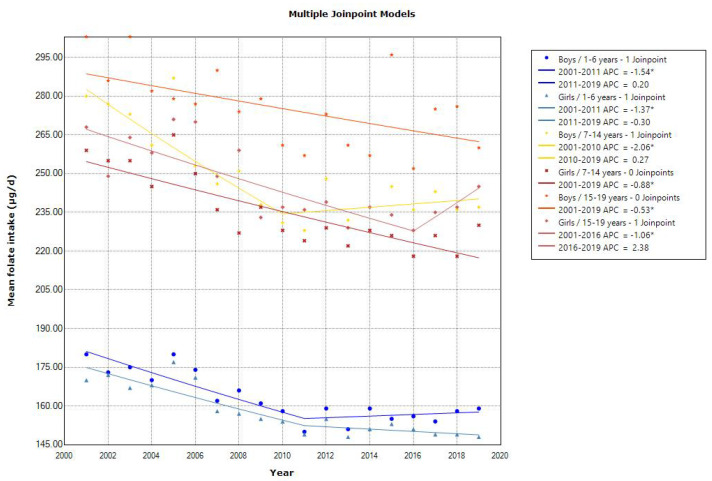
Trends in the proportion of folate intake according to sex and age categories. APC, annual percentage change. * *p* < 0.05.

The trends in mean vitamin and mineral intakes according to sex and age are shown in Table 7, Table 8, Table 9, Table 10, Table 11 and Table 12. Joinpoint regression analyses showed that all age groups showed a declining trend in the mean vitamin A intake, and the slope of the decrease was different after 2006 or 2007 (Figure 6). The 2019 mean vitamin A intake was almost within the estimated average requirement (EAR) in the 2020 DRIs (250–350, 300–550, and 450–650 μg retinol activity equivalents (RAE)/day for 1–5, 6–14, and 15–19 years, respectively). The trend in the mean vitamin B6 intake was unchanged over time. A declining trend in the mean vitamin B12 intake was observed, and the 2019 mean vitamin B12 intake was above the EAR in the 2020 DRIs (0.8–0.9, 1.1–2.0, and 2.0 μg/day for 1–5, 6–14, and 15–19 years, respectively). Similarly, the mean folate intake showed a decreasing trend in toddlers aged 1–6 years (APC of 1.54 for boys and 1.37 for girls [2001–2011], *p* < 0.001), school children aged 7–14 years (APC of 2.06 for boys [2001–2010] and 0.88 for girls, *p* < 0.001), and adolescents aged 15–19 years (APC of 0.53 for boys, *p* < 0.05 and 1.06 for girls [2001–2016], *p* < 0.001) (Figure 7). However, the 2019 mean folate intake was within or above the recommended dietary allowance (RDA) in the 2020 DRIs (90–110, 140–240, and 240 μg/day for 1–5, 6–14, and 15–19 years, respectively). The mean vitamin D intake showed a decreasing trend in toddler girls and adolescents, and the 2019 mean vitamin D intake was below the adequate intake in the 2020 DRIs, especially in adolescents (8.5–9.0 μg/d for 15–19 years). The mean vitamin K intake also showed a declining trend in toddlers, and the 2019 mean vitamin K intake was above the adequate intake in the 2020 DRIs (50–70, 80–170, and 150–160 μg/day for 1–5, 6–14, and 15–19 years, respectively).

**Figure 8:**
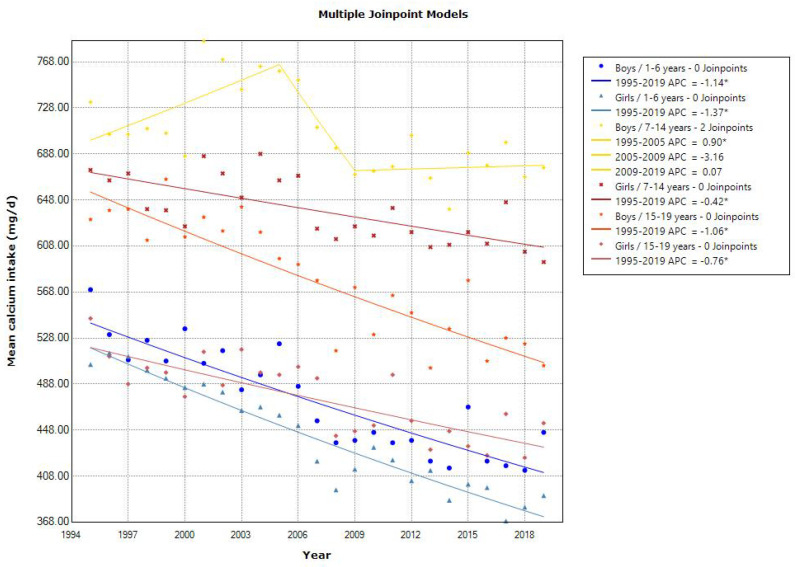
Trends in the proportion of calcium intake according to sex and age categories. APC, annual percentage change. * *p* < 0.05.

**Figure 9:**
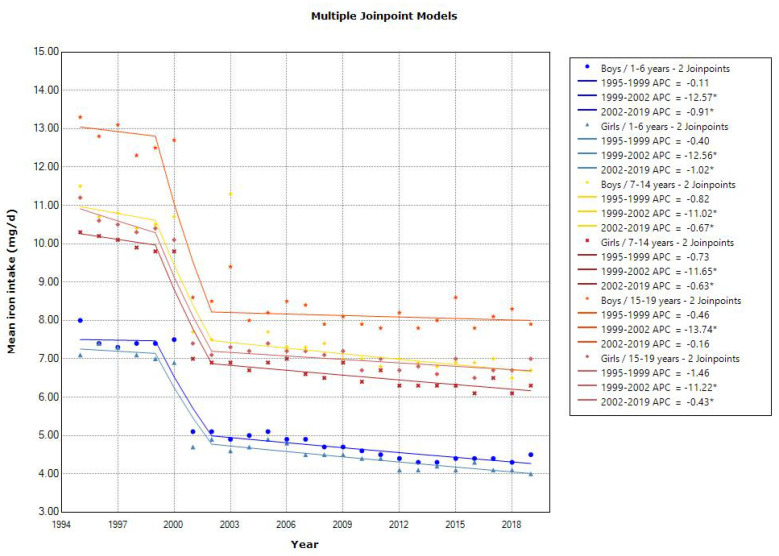
Trends in the proportion of iron intake according to sex and age categories. APC, annual percentage change. * *p* < 0.05.

The mean calcium intake showed a declining trend in toddlers aged 1–6 years (APC of 1.14 for boys and 1.37 for girls, *p* < 0.001), school girls aged 7–14 years (APC of 0.42, *p* < 0.001), and adolescents aged 15–19 years (APC of 1.06 for boys and 0.76 for girls, *p* < 0.001) (Figure 8). The 2019 mean calcium intake in young girls and adolescents was lower than the RDA in the 2020 DRIs (400–600, 550–1000, and 650–800 mg/day for 1–5, 6–14, and 15–19 years, respectively). All age groups showed a decreasing trend in the mean iron intake, with different slopes of a decrease in the three time periods (1995–1999, 1999–2002, and 2002–2019) (Figure 9). The 2019 mean iron intake was within the RDA in the 2020 DRIs (4.5–5.5, 5.5–10.0 (12.0 for menstruating), and 6.5–10.0 (10.5 for menstruating) mg/day for 1–5, 6–14, and 15–19 years, respectively), except for in young girls. The trend in the mean zinc intake was unchanged over time in toddlers and school girls, and the 2019 mean zinc intake was within or above the EAR in the 2020 DRIs (2–3, 3–9, and 7–10 mg/day for 1–5, 6–14, and 15–19 years, respectively). While the trend in the mean copper intake was slightly decreased in all age groups, the 2019 mean copper intake was above the EAR in the 2020 DRIs (0.2–0.3, 0.4–0.7, and 0.6–0.8 mg/day for 1–5, 6–14, and 15–19 years, respectively).

## 4. Discussion

This study showed the trends in energy and nutrient intakes in Japanese children and adolescents for 25 years. The overall trend was a decrease in energy and nutrient intakes, although some nutrient intakes remained unchanged or increased by sex and age group.

The number of survey participants has decreased over the years. This may be linked to the declining trend in the number of live births in Japan, ranging from 1,187,064 in 1995 to 865,239 in 2019 [13]. Moreover, the household response rates are relatively low (63.5%, 2019) [6], although the response rate of individual children and adolescents is unknown. For more detailed analysis of nutrient intake by age group, it would be necessary to increase the response rate of households with children and adolescents in future surveys.

A declining trend in energy intake in boys (young group) and girls (all age groups) was observed in this study, which is consistent with adults [1], while the latest mean energy intake in 2019 was almost within the estimated energy requirement [10]. Moreover, we found that the mean energy intake from protein, fat, and carbohydrates changed little over time. The 2019 mean energy intakes were within the DG, except for the mean energy intake from fat in school and adolescent girls. A previous study showed that a higher household income was associated with higher energy from fat in adolescents [14]. This finding suggested that further study is required to determine the dietary fat source and the underlying factors that contribute to a high fat intake, especially in adolescents.

Although a decreasing trend in the mean salt equivalent in all age groups was observed in this study, the latest mean salt equivalent in 2019 was above the DG. Seasonings, such as soya sauce and soybean paste, account for approximately 70% of the dietary salt source [15]. Therefore, further salt-reduction measures need to be encouraged, especially in adolescent boys. These measures include expanding the options of low-sodium meals in school and university cafeterias, and by warning about salt consumption on food labels at on-campus stores.

Declining trends in mean vitamin A, vitamin B12, folate, and vitamin K intakes were observed, but none of the latest mean intakes in 2019 were deficient compared with the 2020 DRIs. However, the mean vitamin D intake decreased over time and was below the adequate intake, especially in adolescents. Because vitamin D deficiency can cause rickets and osteomalacia, high-risk adolescents may need to incorporate sunlight exposure and dietary modification with vitamin D supplements and vitamin D-fortified foods into their daily lives [16]. With regard to the decrease in vitamin A intake, it should be noted that prior to 2000, the International Unit (IU) was used as the unit of vitamin A intake.

A declining trend in the mean calcium intake was observed in all age groups, except for in school boys aged 7–14 years. Our finding that the mean calcium intake in young girls and adolescents was lower than the RDA is alarming. However, we observed trends in calcium intake from foods and did not consider calcium intake from breast milk or formula. Breast milk or formula may be able to compensate for this deficiency in calcium intake. Therefore, continued breastfeeding after the age of 1 year is recommended in Japan, in alignment with the World Health Organization’s recommendation to continue breastfeeding until the age of 2 years and older [17]. Further research is required to assess the overall calcium intake, including breast milk and formula, especially for 1–2-year-olds.

A decreasing trend in the mean iron intake was found in all age groups, and the 2019 mean iron intake was within the RDA, except for that in young girls. The revisions to the Standard Tables of Food Composition in Japan (4th edition for the 1995–2000 survey and 5th edition for the 2001–2004 survey) may have affected the changes in iron intake since 2001. The World Health Organization warns that the global prevalence of anemia in children aged 6–59 months was 39.8% in 2019 [18], although the actual status of iron deficiency anemia in young Japanese girls is unclear. Therefore, more public health attention is required regarding iron intake and the risks associated with iron deficiency in young girls.

This study has several limitations. First, dietary intake assessed by self-administered dietary records might not represent long-term habitual intake. Second, certain groups may have underreported their dietary intake, because the underreporting of energy intake in young children (1–5 years) and adolescents (15–19 years) and in children with obesity has previously been reported [19]. Third, although three age categories are insufficient because of children’s growth, nutrient intakes in the detailed age categories were unavailable in the survey reports from which the data were obtained. Fourth, while many nutrient intakes may have declining trends because the energy intake has decreased over the years, the survey reports do not disclose the values per 1000 kcal. Fifth, this study did not include nutrient intake from breast milk or formula in 1–2-year-olds. The National Nutrition Survey on Preschool Children showed that the percentage of children weaned by 12 months of age was 54.4% in 2005 and 34.7% in 2015 [20]. Sixth, children and adolescents with various illnesses requiring specific dietary regimens such as food allergies and diabetes could not be excluded from this study because the survey did not collect this information. Although these limitations should be noted when interpreting the results, this study showed the annual trends in mean energy and nutrient intakes using nationally representative data for Japanese children and adolescents over the last 25 years.

## 5. Conclusions

We found declining trends, with some exceptions, in energy and some nutrient intakes in Japanese children and adolescents from 1995 to 2019. Continuous monitoring of the dietary intake and further research are required to raise awareness of unhealthy diet habits and to improve the food environment for the healthy growth and development of children and adolescents.

## Figures and Tables

**Table 1:** Trends in energy and nutrient intakes in young boys aged 1–6 years in 1995–2019.

Variables	1995	1996	1997	1998	1999	2000	2001	2002	2003	2004	2005	2006	2007	2008	2009	2010	2011	2012	2013	2014	2015	2016	2017	2018	2019	*p* Trend
*n*	490	448	381	475	377	361	396	346	347	263	229	276	243	204	238	236	209	846	197	181	182	611	197	181	105	
Energy (kcal/d)																										
Mean	1530	1450	1435	1428	1372	1428	1421	1383	1337	1342	1348	1353	1389	1346	1303	1309	1316	1295	1275	1308	1361	1280	1302	1268	1304	<0.001
SD	691	414	413	427	395	442	419	415	396	391	390	396	416	393	351	396	378	377	382	360	405	315	373	332	329	
Protein (g/d)																										
Mean	56.9	53.7	53.6	54.1	51.7	53.7	49.6	49.1	47.0	47.4	48.9	47.6	48.8	46.0	45.6	45.6	46.1	44.6	44.5	45.0	46.7	45.1	44.3	45.4	47.2	<0.001
SD	23.0	18.4	18.8	19.6	17.4	19.0	17.6	17.4	16.5	16.4	17.0	16.5	17.4	14.3	14.6	16.2	15.9	15.7	15.6	15.1	15.4	13.5	15.3	15.7	15.9	
Total fat (g/d)																										
Mean	49.5	47.5	46.3	45.5	44.4	46.3	45.8	44.3	43.6	43.2	42.5	44.0	43.8	41.8	40.6	42.9	43.1	40.7	40.3	42.8	43.5	40.9	41.1	39.9	43.2	<0.001
SD	21.7	20.0	19.0	18.8	17.4	19.2	18.8	18.2	18.2	17.8	16.6	18.0	19.1	17.6	15.5	19.1	18.7	17.4	18.3	18.4	18.5	15.8	17.5	16.4	16.8	
Saturated fat (g/d)																										
Mean	17.00	16.32	15.69	15.51	14.92	15.82	−	13.87	13.62	−	13.66	14.49	13.64	12.97	12.79	13.54	13.65	13.05	12.69	13.26	14.19	12.80	12.69	13.31	14.92	0.001
SD	8.76	7.57	6.90	7.17	6.37	7.37	−	6.66	6.15	−	6.00	7.07	6.95	6.19	5.86	6.61	6.65	6.38	6.50	6.65	7.09	5.55	6.23	6.14	6.54	
Cholesterol (mg/d)																										
Mean	275	275	259	258	249	260	260	265	245	243	220	223	244	219	224	217	229	212	209	212	209	206	196	197	206	<0.001
SD	182	161	143	153	144	158	152	159	177	151	133	122	147	132	131	125	142	135	132	129	115	118	110	126	131	
Carbohydrate (g/d)																										
Mean	212.7	200.4	199.4	198.9	190.2	197.6	199.3	193.6	186.2	187.7	189.2	187.4	195.9	192.1	185.1	181.7	182.1	184.0	180.0	182.0	191.5	179.4	184.9	177.7	177.5	<0.001
SD	122.7	59.3	59.2	59.2	58.8	63.3	60.2	59.6	56.3	57.5	53.5	58.2	60.1	56.6	52.0	53.0	50.4	53.4	51.2	47.3	57.4	44.7	52.1	47.1	41.5	
Total dietary fiber (g/d)																										
Mean	−	−	−	−	−	−	9.1	8.8	8.5	8.5	9.8	9.0	9.0	9.1	8.7	8.9	8.4	8.7	8.6	8.6	9.0	8.6	8.7	8.8	11.5	0.687
SD	−	−	−	−	−	−	4.2	3.8	4.0	3.8	4.3	4.2	3.8	3.9	3.6	3.9	3.5	3.8	3.7	3.1	3.7	3.2	3.4	3.8	3.7	
Sodium (mg/d)																										
Mean	3141	2927	2984	2951	2864	2880	−	2536	2411	−	2452	2472	2558	2372	2517	2360	2327	2262	2171	2215	2184	2097	2101	2153	2108	<0.001
SD	1589	1228	1406	1375	1470	1258	−	1224	1070	−	945	1105	1006	950	1043	944	993	937	838	888	818	735	791	1189	834	
Salt (g/d)																										
Mean	8.0	7.4	7.6	7.5	7.3	7.3	6.6	6.4	6.1	5.9	6.2	6.3	6.5	6.0	6.4	6.0	5.9	5.7	5.5	5.6	5.5	5.3	5.3	5.5	5.4	<0.001
SD	4.0	3.1	3.6	3.5	3.7	3.2	3.0	3.1	2.7	2.6	2.4	2.8	2.6	2.4	2.6	2.4	2.5	2.4	2.1	2.3	2.1	1.9	2.0	3.0	2.1	
Energy from carbohydrate (%energy)																										
Mean	56.2	56.2	56.5	56.7	56.0	56.2	57.4	57.4	57.0	57.3	57.6	57.1	58.2	58.9	58.3	57.3	57.3	58.6	58.5	57.7	58.0	57.8	58.7	58.1	56.4	0.002
SD	7.8	7.9	7.9	7.7	7.5	7.5	7.9	7.9	7.6	7.9	6.5	7.9	8.5	7.4	7.0	7.7	7.9	7.9	8.5	7.9	7.4	7.3	7.6	9.0	7.2	
Energy from protein (%energy)																										
Mean	14.9	14.8	14.9	15.2	15.2	15.1	−	14.2	14.1	−	14.5	14.1	14.0	13.8	14.1	13.9	14.0	13.7	13.9	13.7	13.8	14.1	13.5	14.2	14.4	0.001
SD	2.7	2.9	2.7	3.1	3.3	3.1	−	2.6	2.6	−	2.4	2.8	2.8	2.4	2.8	2.7	2.6	2.6	2.5	2.4	2.5	2.4	2.5	2.6	2.6	
Energy from fat (%energy)																										
Mean	28.9	29.0	28.6	28.1	28.8	28.7	28.6	28.4	28.9	28.6	27.9	28.8	27.8	27.4	27.7	28.8	28.7	27.7	27.5	28.6	28.2	28.1	27.7	27.8	29.2	0.061
SD	6.7	7.0	7.1	6.5	6.6	6.3	6.9	7.0	6.8	7.1	5.7	6.9	7.5	6.7	6.2	6.9	6.7	7.1	7.5	7.2	6.5	6.4	6.9	7.6	6.2	

The intake of total dietary fiber has been reported since 2001. The intakes of saturated fat, sodium, and energy from protein in 2001 and 2004 were not described in the respective survey reports.

**Table 2:** Trends in energy and nutrient intakes in young girls aged 1–6 years in 1995–2019.

Variables	1995	1996	1997	1998	1999	2000	2001	2002	2003	2004	2005	2006	2007	2008	2009	2010	2011	2012	2013	2014	2015	2016	2017	2018	2019	*p* Trend
*n*	501	437	363	386	372	343	401	341	292	257	234	283	245	217	229	225	185	799	177	164	171	633	176	208	130	
Energy (kcal/d)																										
Mean	1363	1428	1377	1363	1338	1326	1317	1307	1261	1319	1328	1245	1270	1176	1207	1212	1242	1215	1209	1184	1200	1237	1198	1184	1201	<0.001
SD	437	426	459	391	396	368	416	389	364	349	392	330	393	339	347	339	378	333	356	342	291	333	343	331	332	
Protein (g/d)																										
Mean	51.0	52.7	51.4	51.1	50.2	50.6	46.6	47.2	44.2	45.6	47.4	44.7	44.7	41.9	42.3	42.6	43.5	42.2	42.8	41.0	42.4	43.1	41.3	42.0	42.5	<0.001
SD	18.2	17.9	18.7	16.5	17.3	16.3	16.6	16.5	13.9	13.7	15.6	14.2	16.7	15.3	14.6	14.5	15.7	13.7	14.3	13.0	13.1	13.8	14.9	14.2	13.7	
Total fat (g/d)																										
Mean	44.7	47.3	46.1	43.7	44.0	43.9	42.0	41.8	40.2	42.2	42.5	39.9	42.2	37.3	38.6	38.8	39.6	37.8	38.4	38.0	37.0	38.9	38.4	37.9	38.5	<0.001
SD	20.0	19.3	21.3	17.5	18.2	18.0	17.8	17.0	17.0	15.7	17.7	15.7	18.4	16.1	15.7	14.9	17.2	15.2	15.8	16.2	14.1	15.3	19.5	16.1	15.8	
Saturated fat (g/d)																										
Mean	15.33	16.05	15.62	14.90	14.66	14.80	−	13.18	12.73	−	13.43	12.60	13.23	11.58	12.20	12.36	12.76	12.14	12.22	11.62	11.41	12.20	11.83	12.76	13.33	0.001
SD	7.72	7.51	7.68	6.77	6.51	6.65	−	6.02	6.18	−	6.69	5.96	6.40	5.58	5.68	5.64	5.87	5.81	5.71	5.97	5.20	5.63	7.00	6.30	6.71	
Cholesterol (mg/d)																										
Mean	254	273	267	257	245	255	247	242	222	255	228	225	234	201	202	208	222	198	204	189	203	200	194	191	174	<0.001
SD	147	151	158	149	140	135	148	139	130	144	128	133	143	134	117	127	136	115	120	112	126	122	114	132	115	
Carbohydrate (g/d)																										
Mean	187.9	196.4	188.0	190.2	184.2	180.4	184.9	182.6	177.8	185.7	185.3	173.1	174.7	165.4	169.9	170.1	174.1	173.2	169.7	166.4	170.7	175.2	168.2	165.2	167.5	<0.001
SD	59.9	61.3	61.7	56.6	56.6	50.1	59.6	55.3	50.3	53.1	55.6	47.3	55.8	46.1	49.8	49.1	52.4	46.8	49.8	46.9	40.3	46.2	40.5	46.5	43.4	
Total dietary fiber (g/d)																										
Mean	−	−	−	−	−	−	8.6	8.7	8.4	8.3	9.4	9.0	8.3	8.3	8.5	8.7	8.3	8.5	8.3	8.1	8.4	8.6	8.2	8.2	10.6	0.248
SD	−	−	−	−	−	−	3.8	3.7	3.4	3.2	4.1	3.7	3.4	3.8	3.7	4.2	3.4	3.4	3.5	3.2	3.3	3.3	3.7	3.4	3.6	
Sodium (mg/d)																										
Mean	2734	2999	2847	2813	2679	2738	−	2518	2289	−	2341	2319	2320	2277	2263	2145	2230	2103	1973	1955	2059	2134	2046	1980	1962	<0.001
SD	1306	1356	1262	1235	1155	1107	−	1135	998	−	1004	893	978	977	990	913	888	865	786	761	792	803	860	790	751	
Salt (g/d)																										
Mean	6.9	7.6	7.2	7.1	6.8	7.0	6.2	6.4	5.8	5.7	5.9	5.9	5.9	5.8	5.7	5.4	5.7	5.3	5.0	5.0	5.2	5.4	5.2	5.0	5.0	<0.001
SD	3.3	3.4	3.2	3.1	2.9	2.8	2.7	2.9	2.5	2.3	2.6	2.3	2.5	2.5	2.5	2.3	2.3	2.2	2.0	1.9	2.0	2.0	2.2	2.0	1.9	
Energy from carbohydrate (%energy)																										
Mean	56.2	55.8	55.6	56.5	55.9	55.5	57.6	57.3	57.8	57.7	57.5	57.2	56.8	58.0	57.8	57.6	57.8	58.7	57.8	57.9	58.6	58.5	58.8	57.7	57.7	<0.001
SD	7.7	7.2	7.5	7.1	7.6	7.8	7.4	7.3	7.0	7.3	7.7	7.7	8.7	8.2	8.0	7.2	7.6	7.7	7.1	7.2	7.0	6.9	8.6	8.3	6.3	
Energy from protein (%energy)																										
Mean	15.0	14.8	15.0	15.1	15.1	15.3	−	14.5	14.1	−	14.3	14.4	14.0	14.2	14.0	14.0	14.1	13.9	14.1	13.9	14.0	13.9	13.7	14.2	14.1	<0.001
SD	3.0	2.8	2.6	2.7	3.1	3.1	−	2.7	2.4	−	2.8	2.9	3.0	2.8	2.9	2.8	2.9	2.5	2.5	2.4	2.3	2.3	2.6	2.8	2.3	
Energy from fat (%energy)																										
Mean	28.7	29.4	29.4	28.4	29.1	29.2	28.2	28.2	28.1	28.4	28.2	28.4	29.2	27.9	28.2	28.4	28.1	27.4	28.1	28.2	27.4	27.6	27.5	28.1	28.2	<0.001
SD	6.8	6.6	6.8	6.3	6.6	6.5	6.5	6.7	6.4	6.4	6.5	6.6	7.6	7.0	6.8	6.2	6.6	6.8	6.5	6.7	6.2	6.2	7.7	7.2	6.0	

The intake of total dietary fiber has been reported since 2001. The intakes of saturated fat, sodium, and energy from protein in 2001 and 2004 were not described in the respective survey reports.

**Table 3:** Trends in energy and nutrient intakes in boys aged 7–14 years in 1995–2019.

Variables	1995	1996	1997	1998	1999	2000	2001	2002	2003	2004	2005	2006	2007	2008	2009	2010	2011	2012	2013	2014	2015	2016	2017	2018	2019	*p* Trend
*n*	803	690	647	705	597	602	591	452	472	419	365	422	392	367	377	390	352	1271	314	320	315	1045	267	273	250	
Energy (kcal/d)																										
Mean	2165	2108	2122	2090	2078	2058	2196	2172	2148	2154	2152	2080	2103	2065	2032	2040	2018	2111	2059	2107	2087	2100	2132	2042	2047	0.033
SD	580	556	556	530	541	532	663	601	686	567	547	509	555	527	538	511	521	519	546	570	574	549	584	603	551	
Protein (g/d)																										
Mean	84.4	80.8	81.7	80.8	80.6	79.5	80.6	79.0	77.9	77.2	78.3	76.5	75.3	74.1	72.9	73.5	71.8	76.2	74.8	74.7	74.2	75.4	76.3	73.4	74.3	<0.001
SD	25.2	22.6	23.8	24.6	23.1	22.4	27.3	23.7	25.0	22.2	21.8	20.6	21.9	19.7	19.9	21.0	19.8	20.3	22.0	24.6	22.0	20.0	23.8	23.7	20.8	
Total fat (g/d)																										
Mean	72.5	70.2	72.2	70.1	69.5	68.7	69.5	70.3	68.2	68.2	68.4	68.5	69.6	66.1	66.5	66.4	65.7	68.7	66.8	68.0	69.2	66.9	70.6	67.2	67.4	0.005
SD	25.9	25.0	26.0	24.2	24.5	23.5	26.5	26.2	26.8	24.9	24.8	22.0	26.1	22.6	25.5	22.2	22.0	23.8	23.1	25.7	23.3	22.2	25.3	26.4	23.4	
Saturated fat (g/d)																										
Mean	24.97	24.38	24.98	24.51	24.17	23.75	−	22.23	21.30	−	22.25	22.37	21.91	21.12	20.98	21.15	21.11	22.17	21.24	20.61	22.15	21.36	22.07	23.15	23.27	0.007
SD	9.71	9.20	9.61	9.11	9.54	8.63	−	9.22	8.63	−	9.03	8.45	8.68	7.96	8.49	7.37	7.62	8.43	7.93	8.17	7.99	7.65	8.61	9.42	9.07	
Cholesterol (mg/d)																										
Mean	400	386	396	390	382	384	382	390	383	354	366	363	352	354	335	333	333	341	329	332	334	320	317	327	324	<0.001
SD	206	205	200	193	199	201	214	188	218	184	192	173	171	179	170	161	176	186	191	186	162	149	183	179	183	
Carbohydrate (g/d)																										
Mean	289.1	283.7	281.9	278.9	277.5	275.9	305.4	299.2	298.6	301.0	298.4	281.8	286.1	286.0	278.0	279.1	276.9	288.8	281.7	290.4	283.9	289.9	289.5	277.5	277.2	0.58
SD	84.3	80.4	79.1	76.0	78.6	77.2	93.1	88.9	101.9	84.7	77.3	77.0	81.2	78.4	78.7	80.2	79.0	76.3	82.3	81.9	88.6	85.7	83.0	86.2	81.6	
Total dietary fiber (g/d)																										
Mean	−	−	−	−	−	−	14.8	14.3	14.1	14.0	14.6	13.7	13.9	13.8	13.4	12.9	13.0	13.7	13.4	13.3	13.9	13.4	14.1	13.0	18.1	0.158
SD	−	−	−	−	−	−	5.2	5.0	5.2	4.8	4.7	5.5	5.1	4.9	4.9	4.2	4.5	4.6	4.6	4.2	5.2	4.6	5.0	4.4	5.6	
Sodium (mg/d)																										
Mean	4708	4680	4609	4530	4477	4367	−	4053	3945	−	3888	3885	3850	3787	3760	3665	3737	3679	3583	3648	3583	3600	3532	3499	3515	<0.001
SD	1804	1905	2203	1646	1563	1538	−	1443	1703	−	1308	1315	1420	1313	1273	1241	1257	1220	1142	1238	1279	1218	1112	1212	1143	
Salt (g/d)																										
Mean	12.0	11.9	11.7	11.5	11.4	11.1	10.5	10.3	10.0	9.6	9.9	9.9	9.8	9.6	9.5	9.3	9.5	9.3	9.1	9.3	9.1	9.1	9.0	8.9	8.9	<0.001
SD	4.6	4.8	5.6	4.2	4.0	3.9	4.1	3.7	4.3	3.3	3.3	3.3	3.6	3.3	3.2	3.2	3.2	3.1	2.9	3.1	3.2	3.1	2.8	3.1	2.9	
Energy from carbohydrate (%energy)																										
Mean	54.4	54.8	54.2	54.5	54.5	54.6	57.1	56.4	57.0	57.3	57.1	55.6	56.1	57.0	56.4	56.2	56.4	56.5	56.3	57.0	55.8	56.8	56.0	56.2	55.9	0.11
SD	6.6	6.1	6.5	6.6	6.6	6.2	5.9	6.7	6.3	6.6	6.1	6.5	7.3	6.1	6.9	6.7	6.4	6.6	6.9	6.6	6.5	6.2	6.2	6.8	6.3	
Energy from protein (%energy)																										
Mean	15.7	15.4	15.5	15.5	15.6	15.6	−	14.6	14.6	−	14.6	14.8	14.4	14.5	14.5	14.5	14.4	14.5	14.6	14.2	14.3	14.5	14.3	14.5	14.6	<0.001
SD	2.7	2.4	2.6	2.7	2.7	2.5	−	2.2	2.2	−	2.2	2.4	2.3	2.2	2.4	2.6	2.4	2.3	2.3	2.3	2.4	2.2	2.2	2.5	2.4	
Energy from fat (%energy)																										
Mean	29.9	29.7	30.4	30.0	29.8	29.8	28.2	28.9	28.4	28.3	28.3	29.6	29.5	28.6	29.0	29.2	29.2	29.0	29.1	28.8	29.8	28.7	29.6	29.4	29.5	0.211
SD	6.0	5.7	6.1	5.9	5.7	5.6	5.4	6.3	5.7	5.9	5.6	5.7	6.5	5.7	6.3	6.2	5.6	5.9	6.2	5.9	5.9	5.5	5.9	5.8	5.8	

The intake of total dietary fiber has been reported since 2001. The intakes of saturated fat, sodium, and energy from protein in 2001 and 2004 were not described in the respective survey reports.

**Table 4:** Trends in energy and nutrient intakes in girls aged 7–14 years in 1995–2019.

Variables	1995	1996	1997	1998	1999	2000	2001	2002	2003	2004	2005	2006	2007	2008	2009	2010	2011	2012	2013	2014	2015	2016	2017	2018	2019	*p* Trend
*n*	739	668	628	675	535	581	580	464	467	351	376	393	403	337	382	349	368	1285	295	300	282	943	245	244	204	
Energy (kcal/d)																										
Mean	1931	1923	1901	1878	1874	1843	1913	1926	1877	1903	1870	1905	1871	1794	1842	1817	1853	1844	1818	1811	1825	1837	1903	1785	1820	0.001
SD	564	474	455	446	432	448	497	503	528	427	456	433	466	451	399	417	422	409	422	366	470	387	425	415	455	
Protein (g/d)																										
Mean	76.3	74.4	74.7	73.8	72.8	72.0	71.1	69.9	69.3	69.8	69.1	70.9	67.7	65.4	68.0	65.7	67.0	66.5	66.4	66.3	65.1	65.8	67.6	65.6	68.1	<0.001
SD	22.3	20.1	19.5	20.7	20.1	19.2	20.6	19.4	21.3	17.6	18.5	18.9	17.7	17.9	17.5	17.7	17.2	16.0	16.3	15.7	18.9	15.1	18.0	17.9	17.0	
Total fat (g/d)																										
Mean	65.0	65.8	66.4	63.3	64.1	63.5	62.7	61.9	61.4	60.8	60.2	64.5	63.2	59.5	60.8	59.1	62.5	61.2	60.8	59.4	59.7	60.5	63.1	59.8	62.1	0.001
SD	21.5	22.9	23.6	21.6	22.0	21.7	22.9	22.8	24.7	18.7	21.0	20.8	22.0	21.8	20.8	18.9	20.7	19.4	20.4	18.6	20.7	18.5	20.0	20.7	23.3	
Saturated fat (g/d)																										
Mean	22.03	22.59	22.69	21.57	21.76	21.25	−	19.21	18.98	−	19.30	20.40	20.28	19.13	19.26	19.20	20.29	19.49	19.50	18.77	19.11	19.24	20.01	20.39	21.13	0.017
SD	8.50	8.88	8.53	8.02	8.01	7.73	−	7.90	8.00	−	7.28	7.12	8.09	7.47	7.40	6.76	7.15	6.79	7.10	6.59	7.49	6.40	6.81	7.62	8.87	
Cholesterol (mg/d)																										
Mean	368	370	375	356	358	341	348	358	340	322	331	347	334	318	327	298	315	298	296	301	308	290	306	292	304	<0.001
SD	199	194	193	186	181	172	168	169	184	150	176	187	169	174	166	163	169	144	145	154	168	140	159	154	161	
Carbohydrate (g/d)																										
Mean	256.9	255.3	248.3	249.7	248.2	242.5	261.1	267.1	256.5	264.0	257.7	253.7	251.6	243.2	249.9	249.2	249.0	250.4	245.2	246.7	250.1	250.6	259.1	239.2	240.2	0.102
SD	98.3	67.8	63.3	65.5	62.2	62.5	69.1	71.6	73.4	64.9	66.7	62.9	69.3	66.7	59.1	62.9	59.8	63.4	62.8	54.7	68.4	59.3	62.9	57.3	61.7	
Total dietary fiber (g/d)																										
Mean	−	−	−	−	−	−	13.7	13.3	13.3	13.5	14.1	13.1	12.7	12.8	13.0	12.4	12.6	12.9	12.6	12.8	12.6	12.6	13.0	12.2	16.6	0.032
SD	−	−	−	−	−	−	4.7	4.7	4.9	4.2	4.8	4.3	4.5	5.2	4.5	4.1	4.5	4.4	4.5	3.7	4.6	3.9	4.9	3.9	5.3	
Sodium (mg/d)																										
Mean	4385	4309	4358	4312	4109	4097	−	3623	3657	−	3638	3721	3558	3355	3562	3395	3526	3383	3316	3291	3242	3249	3262	3245	3216	<0.001
SD	1819	1549	1616	1593	1427	1422	−	1248	1430	−	1200	1229	1264	1177	1136	1095	1156	1165	1032	961	1233	953	1061	1007	1034	
Salt (g/d)																										
Mean	11.1	10.9	11.1	11.0	10.4	10.4	9.7	9.2	9.3	9.3	9.2	9.5	9.0	8.5	9.0	8.6	9.0	8.6	8.4	8.4	8.2	8.3	8.3	8.2	8.2	<0.001
SD	4.6	3.9	4.1	4.0	3.6	3.6	3.5	3.2	3.6	3.2	3.0	3.1	3.2	3.0	2.9	2.8	2.9	3.0	2.6	2.4	3.1	2.4	2.7	2.6	2.6	
Energy from carbohydrate (%energy)																										
Mean	53.9	54.0	53.1	54.1	53.9	53.6	55.9	56.9	56.1	56.6	56.4	54.8	55.2	55.8	55.8	56.4	55.4	55.8	55.5	56.0	56.5	56.1	56.1	55.4	54.6	0.044
SD	6.6	6.4	6.3	6.5	6.7	6.3	6.3	6.4	6.5	5.8	6.3	6.6	6.5	7.3	7.3	6.1	6.3	6.5	6.9	6.5	6.4	6.2	6.3	6.9	6.2	
Energy from protein (%energy)																										
Mean	15.9	15.6	15.8	15.8	15.6	15.7	−	14.6	14.8	−	14.9	14.9	14.6	14.7	14.8	14.5	14.6	14.5	14.7	14.7	14.3	14.5	14.3	14.7	15.1	0.001
SD	2.8	2.6	2.6	2.8	2.6	2.6	−	2.4	2.4	−	2.4	2.3	2.4	2.4	2.6	2.3	2.4	2.3	2.4	2.3	2.1	2.4	2.3	2.2	2.3	
Energy from fat (%energy)																										
Mean	30.2	30.5	31.1	30.1	30.4	30.6	29.1	28.5	29.0	28.7	28.7	30.3	30.2	29.5	29.3	29.1	30.1	29.7	29.8	29.2	29.2	29.4	29.6	29.9	30.2	0.233
SD	5.9	5.6	5.9	6.0	6.1	5.7	5.9	5.9	6.0	5.3	5.5	6.1	6.0	6.5	6.4	5.6	5.6	5.9	6.1	6.0	5.7	5.5	6.1	6.1	6.0	

The intake of total dietary fiber has been reported since 2001. The intakes of saturated fat, sodium, and energy from protein in 2001 and 2004 were not described in the respective survey reports.

**Table 5:** Trends in energy and nutrient intakes in adolescent boys aged 15–19 years in 1995–2019.

Variables	1995	1996	1997	1998	1999	2000	2001	2002	2003	2004	2005	2006	2007	2008	2009	2010	2011	2012	2013	2014	2015	2016	2017	2018	2019	*p* Trend
*n*	500	473	446	416	373	339	358	301	272	239	218	239	201	190	206	193	193	702	175	173	165	559	141	143	130	
Energy (kcal/d)																										
Mean	2589	2489	2557	2439	2431	2407	2545	2500	2533	2431	2452	2508	2440	2380	2481	2555	2439	2504	2510	2367	2641	2425	2486	2527	2515	0.889
SD	795	729	814	702	749	671	885	773	792	654	780	732	733	693	757	823	657	673	751	756	730	640	693	730	780	
Protein (g/d)																										
Mean	99.5	98.1	99.4	94.2	95.1	95.3	91.5	88.9	91.2	85.7	86.7	88.4	87.5	82.3	85.5	86.0	82.9	86.1	85.6	83.0	92.6	85.7	90.1	91.4	88.7	0.005
SD	32.7	31.3	35.0	29.2	33.5	30.8	33.4	29.3	30.6	26.6	27.9	28.8	28.2	24.4	28.7	30.5	25.3	25.5	27.6	26.7	29.5	26.4	27.8	35.8	30.7	
Total fat (g/d)																										
Mean	83.3	80.6	81.8	80.7	79.5	78.2	80.0	79.8	81.8	78.0	77.7	78.3	78.2	73.6	79.1	80.1	74.8	81.2	77.0	77.1	87.0	76.6	79.9	82.3	84.4	0.61
SD	35.4	33.3	35.0	32.5	32.7	30.6	35.4	34.1	32.3	29.1	32.7	30.0	33.4	29.5	34.0	36.9	27.7	31.6	30.3	29.8	32.3	27.1	30.4	33.4	34.2	
Saturated fat (g/d)																										
Mean	27.11	26.47	26.29	26.32	26.54	25.61	−	22.30	22.76	−	22.17	21.91	22.24	21.08	22.53	22.85	21.23	22.87	21.45	21.40	24.55	21.94	21.91	24.92	26.31	0.018
SD	12.78	12.60	12.61	11.95	12.74	11.53	−	11.31	10.04	−	11.13	10.01	11.29	8.93	11.18	12.10	9.38	10.05	9.58	9.62	10.95	9.15	10.11	10.27	12.30	
Cholesterol (mg/d)																										
Mean	490	501	485	476	479	487	463	460	490	433	436	449	467	420	442	439	422	434	407	437	458	431	452	454	474	0.003
SD	263	256	274	251	250	270	236	232	273	195	251	213	246	217	244	240	227	211	213	227	230	212	229	280	284	
Carbohydrate (g/d)																										
Mean	352.2	335.2	347.6	326.1	328.0	322.7	352.1	344.8	345.3	334.8	339.8	349.4	333.6	334.4	344.5	358.8	346.1	344.3	355.2	322.6	358.6	334.6	338.2	340.9	335.2	0.827
SD	115.6	105.3	115.0	105.2	109.0	96.3	130.8	117.3	115.1	95.3	122.6	112.8	109.6	108.3	111.3	120.2	106.6	103.9	117.3	118.5	115.6	99.4	108.3	99.7	113.1	
Total dietary fiber (g/d)																										
Mean	−	−	−	−	−	−	14.4	13.7	14.1	13.4	14.2	14.3	13.7	13.6	13.8	13.7	13.5	14.1	13.9	13.5	15.4	13.5	13.9	14.4	20.0	0.581
SD	−	−	−	−	−	−	6.2	5.9	5.9	5.5	6.1	6.0	5.4	5.8	6.3	5.6	5.5	5.3	6.0	6.2	6.7	5.2	6.0	6.2	7.0	
Sodium (mg/d)																										
Mean	5618	5234	5462	5085	5065	5088	−	4650	4750	−	4533	4521	4614	4303	4393	4509	4279	4316	4297	4160	4427	4077	4062	4304	4080	<0.001
SD	3116	2116	2403	2083	2081	2045	−	1792	1815	−	1654	1720	1692	1491	1658	1772	1620	1588	1734	1496	1742	1401	1397	1701	1385	
Salt (g/d)																										
Mean	14.3	13.3	13.9	12.9	12.9	12.9	12.2	11.8	12.1	11.4	11.5	11.5	11.7	10.9	11.2	11.5	10.9	11.0	10.9	10.6	11.2	10.4	10.3	10.9	10.4	<0.001
SD	7.9	5.4	6.1	5.3	5.3	5.2	5.0	4.6	4.6	4.2	4.2	4.4	4.3	3.8	4.2	4.5	4.1	4.0	4.4	3.8	4.4	3.6	3.5	4.3	3.5	
Energy from carbohydrate (%energy)																										
Mean	55.9	55.3	56.0	54.9	55.2	55.3	57.4	57.2	56.7	57.3	57.4	57.8	57.1	58.5	57.7	58.8	58.8	57.2	58.6	56.5	56.2	57.4	56.6	56.5	56.0	0.057
SD	7.9	7.6	7.7	8.3	8.2	7.4	7.3	8.2	7.9	7.0	8.4	7.5	8.2	7.8	7.5	8.1	7.8	7.8	7.3	7.8	9.3	7.3	8.1	7.9	8.5	
Energy from protein (%energy)																										
Mean	15.6	16.0	15.7	15.6	15.7	15.8	−	14.3	14.5	−	14.4	14.2	14.5	14.0	13.9	13.6	13.7	13.9	13.8	14.2	14.1	14.3	14.6	14.4	14.2	0.003
SD	3.3	3.1	3.2	2.8	2.8	2.8	−	2.6	3.0	−	2.9	3.0	3.0	2.6	2.8	2.6	2.5	2.5	2.6	2.4	2.9	2.9	3.2	2.6	2.6	
Energy from fat (%energy)																										
Mean	28.5	28.7	28.3	29.5	29.1	28.8	28.0	28.4	28.9	28.5	28.2	28.0	28.4	27.5	28.4	27.7	27.5	28.9	27.6	29.3	29.7	28.3	28.8	29.1	29.8	0.559
SD	6.8	6.6	6.8	7.4	7.1	6.7	6.4	7.3	6.6	6.3	7.5	6.4	6.9	6.9	6.8	7.4	6.7	7.0	6.9	6.9	7.8	6.0	7.0	6.8	7.2	

The intake of total dietary fiber has been reported since 2001. The intakes of saturated fat, sodium, and energy from protein in 2001 and 2004 were not described in the respective survey reports.

**Table 6:** Trends in energy and nutrient intakes in adolescent girls aged 15–19 years in 1995–2019.

Variables	1995	1996	1997	1998	1999	2000	2001	2002	2003	2004	2005	2006	2007	2008	2009	2010	2011	2012	2013	2014	2015	2016	2017	2018	2019	*p* Trend
*n*	440	438	418	411	372	369	330	314	291	196	211	219	192	170	197	193	187	599	162	182	169	491	142	134	119	
Energy (kcal/d)																										
Mean	1943	1906	1885	1905	1900	1884	1918	1858	1878	1899	1916	1852	1873	1780	1790	1829	1820	1828	1803	1776	1854	1773	1885	1820	1896	0.002
SD	556	562	496	491	549	509	559	512	517	518	512	480	541	433	516	447	501	477	497	474	460	486	537	418	464	
Protein (g/d)																										
Mean	78.9	74.7	74.5	73.9	74.9	72.2	72.4	68.6	69.5	72.5	72.0	69.6	68.3	64.3	65.6	65.7	65.8	66.5	66.3	62.7	67.5	64.8	67.6	67.4	71.8	<0.001
SD	25.8	24.4	23.6	21.5	24.6	22.0	24.4	21.4	21.6	23.3	22.0	20.9	22.4	17.3	20.8	20.2	20.5	19.9	21.3	19.3	19.9	19.8	20.1	19.0	21.9	
Total fat (g/d)																										
Mean	65.2	62.9	61.9	63.7	63.2	65.1	63.3	61.5	63.9	63.4	64.9	60.8	65.4	57.8	60.5	63.0	61.8	62.3	62.7	59.0	64.6	61.4	66.4	62.9	67.7	0.709
SD	26.2	27.3	23.4	24.2	25.8	24.6	26.8	24.1	24.7	24.5	23.6	22.4	26.3	21.3	23.6	22.8	23.8	24.0	23.5	22.9	24.6	23.2	26.3	20.6	27.2	
Saturated fat (g/d)																										
Mean	20.39	19.90	19.05	20.15	20.14	20.70	−	16.71	18.07	−	17.97	17.17	18.32	16.14	17.25	18.04	17.97	17.94	17.88	16.56	18.00	17.33	19.55	19.17	20.98	0.158
SD	9.39	9.44	8.04	8.66	9.31	9.03	−	7.55	8.53	−	7.56	7.97	9.26	7.71	7.90	8.01	7.98	7.91	8.18	7.70	8.07	7.18	9.39	7.62	10.50	
Cholesterol (mg/d)																										
Mean	431	402	403	389	400	403	393	397	385	382	398	398	382	363	359	366	349	351	349	344	374	351	363	372	381	<0.001
SD	219	227	219	216	215	225	205	195	225	205	198	201	184	169	173	191	179	184	196	172	205	179	167	188	196	
Carbohydrate (g/d)																										
Mean	257.0	256.6	254.2	255.0	253.5	247.5	257.5	250.5	249.0	251.5	253.9	249.3	245.4	243.4	238.0	241.4	242.8	242.8	236.2	242.1	242.7	232.5	246.3	237.3	241.4	<0.001
SD	79.3	79.3	73.8	72.3	79.0	74.1	75.5	72.8	77.6	75.0	77.1	73.2	72.6	66.6	75.4	67.6	72.4	67.3	76.1	67.7	67.4	68.3	74.8	62.5	56.6	
Total dietary fiber (g/d)																										
Mean	−	−	−	−	−	−	13.1	11.9	12.2	12.0	13.7	13.0	12.6	12.4	11.8	11.7	12.4	12.3	11.9	12.1	12.2	11.3	12.1	12.1	17.0	0.439
SD	−	−	−	−	−	−	5.7	5.1	5.7	5.1	6.3	6.0	5.5	5.2	4.6	5.2	5.4	5.1	5.0	5.0	4.1	4.8	4.9	4.9	5.6	
Sodium (mg/d)																										
Mean	4606	4557	4545	4477	4379	4203	−	3838	3955	−	3896	3770	3727	3708	3617	3532	3678	3561	3419	3349	3495	3283	3393	3449	3451	<0.001
SD	2116	2087	2573	1820	1700	1713	−	1538	1495	−	1496	1246	1363	1327	1410	1181	1359	1213	1308	1224	1282	1194	1153	1060	1128	
Salt (g/d)																										
Mean	11.7	11.6	11.5	11.4	11.1	10.7	10.6	9.7	10.0	9.7	9.9	9.6	9.5	9.4	9.2	9.0	9.3	9.0	8.7	8.5	8.9	8.3	8.6	8.8	8.8	<0.001
SD	5.4	5.3	6.5	4.6	4.3	4.4	4.3	3.9	3.8	3.6	3.8	3.2	3.5	3.4	3.6	3.0	3.5	3.1	3.3	3.1	3.3	3.0	2.9	2.7	2.9	
Energy from carbohydrate (%energy)																										
Mean	54.0	55.1	54.9	54.6	54.6	53.8	55.7	55.7	54.9	54.8	54.5	55.5	54.5	56.4	55.1	54.8	55.1	55.0	54.0	56.4	54.3	54.4	54.3	54.1	53.6	0.271
SD	7.8	8.3	8.1	7.4	8.3	7.7	7.5	7.9	8.8	7.9	8.1	7.8	7.6	7.5	7.5	8.5	7.8	7.5	8.2	7.4	8.4	7.1	7.6	7.8	8.1	
Energy from protein (%energy)																										
Mean	16.3	15.8	15.9	15.6	15.9	15.4	−	14.8	14.8	−	15.1	15.2	14.7	14.6	14.8	14.4	14.6	14.6	14.9	14.2	14.7	14.8	14.5	15.0	15.2	0.002
SD	3.3	3.1	3.2	3.0	3.3	2.8	−	2.8	2.7	−	2.9	3.2	2.7	3.0	3.0	3.0	2.7	2.7	3.4	2.7	3.1	3.0	2.9	3.2	3.1	
Energy from fat (%energy)																										
Mean	29.7	29.2	29.2	29.8	29.5	30.8	29.1	29.5	30.2	29.8	30.3	29.3	30.8	29.0	30.0	30.8	30.3	30.4	31.1	29.5	31.0	30.8	31.2	30.9	31.3	0.001
SD	6.9	7.1	7.2	6.8	7.3	6.9	6.5	7.1	7.9	7.1	6.9	6.8	6.6	6.8	6.4	7.7	6.9	6.9	7.1	6.6	7.4	6.2	6.7	6.5	7.4	

The intake of total dietary fiber has been reported since 2001. The intakes of saturated fat, sodium, and energy from protein in 2001 and 2004 were not described in the respective survey reports.

**Table 7:** Trends in vitamin and mineral intakes in young boys aged 1–6 years in 1995–2019.

Variables	1995	1996	1997	1998	1999	2000	2001	2002	2003	2004	2005	2006	2007	2008	2009	2010	2011	2012	2013	2014	2015	2016	2017	2018	2019	*p* Trend
*n*	490	448	381	475	377	361	396	346	347	263	229	276	243	204	238	236	209	846	197	181	182	611	197	181	105	
Vitamin A (retinol equivalent) (IU/μgRAE) *																										
Mean	1869	1832	1653	1853	1950	1891	745	659	677	680	455	512	419	432	411	413	383	404	412	424	418	397	445	401	356	<0.001
SD	1616	1406	1010	2301	2639	1494	591	468	941	406	327	742	338	326	273	446	256	289	272	364	315	330	609	308	198	
Thiamin (mg)																										
Mean	0.87	0.80	0.79	0.79	0.78	0.83	0.66	0.63	0.60	0.61	0.63	0.63	0.61	0.60	0.61	0.60	0.65	0.57	0.58	0.57	0.60	0.58	0.54	0.59	0.68	<0.001
SD	0.75	0.35	0.33	0.32	0.41	0.57	0.28	0.26	0.24	0.23	0.26	0.44	0.26	0.23	0.33	0.29	1.25	0.25	0.24	0.25	0.24	0.23	0.21	0.22	0.29	
Riboflavin (mg)																										
Mean	1.27	1.19	1.16	1.16	1.16	1.22	0.95	0.95	0.91	0.92	0.92	0.91	0.91	0.83	0.85	0.85	0.86	0.84	0.83	0.80	0.89	0.82	0.84	0.79	0.85	<0.001
SD	0.84	0.50	0.46	0.47	0.58	0.65	0.41	0.42	0.43	0.41	0.42	0.50	0.41	0.33	0.37	0.40	0.41	0.38	0.41	0.36	0.43	0.35	0.47	0.33	0.33	
Niacin Equivalent (mgNE)																										
Mean	−	−	−	−	−	−	16.4	16.2	15.6	15.8	16.6	16.0	16.4	15.4	15.4	15.5	15.3	15.0	15.0	15.1	15.4	15.3	14.9	17.9	18.6	0.182
SD	−	−	−	−	−	−	6.4	6.7	5.8	5.8	6.7	6.5	6.6	5.5	5.4	6.5	5.8	5.5	5.6	5.8	5.7	5.1	5.9	6.6	7.3	
Vitamin B6 (mg)																										
Mean	−	−	−	−	−	−	0.76	0.76	0.73	0.76	0.81	0.75	0.74	0.71	0.73	0.72	0.71	0.70	0.68	0.72	0.74	0.73	0.70	0.74	0.77	0.12
SD	−	−	−	−	−	−	0.32	0.34	0.45	0.46	0.68	0.48	0.34	0.27	0.30	0.31	0.29	0.36	0.26	0.28	0.32	0.28	0.30	0.30	0.29	
Vitamin B12 (µg)																										
Mean	−	−	−	−	−	−	4.2	4.6	4.8	4.1	4.4	4.3	4.2	3.5	3.7	3.6	3.5	3.3	2.9	3.1	3.3	3.5	3.3	3.5	4.4	0.009
SD	−	−	−	−	−	−	3.7	4.4	6.8	3.4	4.3	5.9	4.4	3.3	4.5	4.2	3.4	3.5	2.2	2.8	3.0	3.1	3.2	3.6	8.7	
Folate (µg)																										
Mean	−	−	−	−	−	−	180	173	175	170	180	174	162	166	161	158	150	159	151	159	155	156	154	158	159	0.001
SD	−	−	−	−	−	−	90	76	128	72	79	108	75	66	64	68	60	67	65	63	62	63	78	68	58	
Pantothenic acid (mg)																										
Mean	−	−	−	−	−	−	4.54	4.43	4.25	4.28	4.40	4.27	4.23	4.10	4.06	4.06	4.07	4.03	3.93	3.96	4.18	3.96	3.95	4.06	4.26	0.003
SD	−	−	−	−	−	−	1.72	1.68	1.80	1.56	1.62	1.59	1.65	1.40	1.39	1.47	1.44	1.45	1.55	1.31	1.59	1.32	1.47	1.47	1.42	
Vitamin C (mg)																										
Mean	93	96	86	83	90	80	63	59	62	58	67	54	55	54	59	58	54	55	53	54	54	53	48	56	56	<0.001
SD	97	128	105	91	93	104	41	37	54	38	109	38	54	35	53	42	57	41	39	38	35	35	33	39	34	
Vitamin D (µg)																										
Mean	−	−	−	−	−	−	4.4	4.6	4.4	4.4	4.8	4.2	3.7	3.4	4.5	4.3	4.7	4.2	3.8	4.3	4.6	3.9	3.9	4.4	4.1	0.118
SD	−	−	−	−	−	−	4.8	5.0	5.0	4.2	6.3	5.3	4.0	3.5	5.6	4.6	5.8	4.9	4.0	4.9	5.6	3.9	4.5	5.2	5.1	
Vitamin E (alpha-tocopherol) (mg)																										
Mean	−	−	−	−	−	−	5.9	5.8	5.8	5.6	4.8	4.8	4.9	4.7	4.3	4.5	4.3	4.3	4.1	4.4	4.5	4.4	4.3	4.4	4.2	0.001
SD	−	−	−	−	−	−	2.7	2.7	2.7	2.5	2.3	2.2	2.3	2.3	2.1	2.3	2.0	2.2	2.0	1.9	2.0	2.0	2.1	2.2	1.6	
Vitamin K (µg)																										
Mean	−	−	−	−	−	−	145	144	140	143	143	136	131	134	125	121	122	125	124	120	135	125	119	137	132	0.009
SD	−	−	−	−	−	−	137	124	126	129	123	104	115	113	90	91	101	106	106	88	106	93	106	130	105	
Potassium (mg)																										
Mean	−	−	−	−	−	−	1699	1669	1590	1622	1704	1577	1571	1531	1506	1521	1492	1497	1450	1488	1575	1489	1429	1514	1588	0.004
SD	−	−	−	−	−	−	695	707	609	644	644	603	612	526	498	588	546	567	540	520	594	512	546	585	497	
Calcium (mg)																										
Mean	570	531	509	526	508	536	506	517	483	496	523	486	456	437	439	446	437	439	421	415	468	421	417	413	446	<0.001
SD	339	241	231	275	236	275	251	251	232	255	228	253	223	211	224	217	202	232	224	207	246	202	209	208	207	
Magnesium (mg)																										
Mean	−	−	−	−	−	−	168	169	158	162	172	157	158	154	151	150	149	148	146	147	156	148	145	159	158	0.017
SD	−	−	−	−	−	−	66	66	59	65	64	61	57	54	49	56	54	56	53	47	58	46	51	76	50	
Phosphorus (mg)																										
Mean	−	−	−	−	−	−	784	783	735	744	776	743	744	713	707	710	705	695	681	681	733	692	678	696	728	0.001
SD	−	−	−	−	−	−	296	294	278	288	280	274	271	237	248	268	254	255	261	240	275	229	255	254	249	
Iron (mg)																										
Mean	8.0	7.4	7.3	7.4	7.4	7.5	5.1	5.1	4.9	5.0	5.1	4.9	4.9	4.7	4.7	4.6	4.5	4.4	4.3	4.3	4.4	4.4	4.4	4.3	4.5	<0.001
SD	4.9	2.8	2.7	3.1	3.5	3.6	2.2	2.4	2.6	2.5	2.1	2.3	1.9	1.7	1.7	2.0	1.9	1.9	1.8	1.4	1.7	1.6	1.8	1.6	1.5	
Zinc (mg)																										
Mean	−	−	−	−	−	−	5.9	5.9	5.6	5.7	5.8	5.7	5.8	5.6	5.5	5.5	5.5	5.4	5.4	5.3	5.6	5.5	5.4	5.4	5.7	0.005
SD	−	−	−	−	−	−	2.1	2.1	2.1	2.0	2.0	1.9	2.1	1.7	1.9	1.9	1.8	1.8	1.9	1.6	1.8	1.6	1.7	1.8	1.8	
Copper (mg)																										
Mean	−	−	−	−	−	−	0.77	0.77	0.73	0.74	0.78	0.73	0.75	0.73	0.72	0.70	0.68	0.70	0.68	0.69	0.72	0.69	0.71	0.70	0.71	0.002
SD	−	−	−	−	−	−	0.27	0.28	0.28	0.26	0.30	0.26	0.27	0.24	0.24	0.24	0.22	0.24	0.24	0.21	0.25	0.21	0.25	0.24	0.21	

* Prior to 2000, International Unit (IU) was used as the unit of vitamin A intake. The intakes of niacin equivalent, vitamin B6, vitamin B12, folate, pantothenic acid, vitamin D, vitamin E (alpha-tocopherol), vitamin K, potassium, magnesium, phosphorus, zinc, and copper have been reported since 2001.

**Table 8:** Trends in vitamin and mineral intakes in young girls aged 1–6 years in 1995–2019.

Variables	1995	1996	1997	1998	1999	2000	2001	2002	2003	2004	2005	2006	2007	2008	2009	2010	2011	2012	2013	2014	2015	2016	2017	2018	2019	*P* Trend
*n*	501	437	363	386	372	343	401	341	292	257	234	283	245	217	229	225	185	799	177	164	171	633	176	208	130	
Vitamin A (retinol equivalent) (IU/μgRAE) *																										
Mean	1773	1795	1799	1626	1682	1830	679	668	636	672	446	420	407	381	376	406	411	403	355	371	413	391	456	354	345	<0.001
SD	1822	1565	1947	1279	1143	1759	439	500	425	403	438	368	406	269	239	360	388	258	219	320	346	288	765	253	391	
Thiamin (mg)																										
Mean	0.75	0.81	0.77	0.76	0.75	0.77	0.64	0.63	0.59	0.62	0.66	0.59	0.58	0.55	0.56	0.57	0.56	0.55	0.55	0.52	0.54	0.56	0.51	0.57	0.62	<0.001
SD	0.32	0.47	0.37	0.31	0.32	0.44	0.29	0.28	0.22	0.26	0.76	0.24	0.24	0.26	0.25	0.26	0.26	0.22	0.21	0.20	0.22	0.21	0.26	0.25	0.31	
Riboflavin (mg)																										
Mean	1.12	1.17	1.15	1.14	1.09	1.13	0.91	0.91	0.87	0.90	0.91	0.85	0.82	0.77	0.79	0.82	0.82	0.79	0.80	0.72	0.77	0.79	0.76	0.75	0.76	<0.001
SD	0.49	0.53	0.51	0.48	0.44	0.55	0.38	0.36	0.39	0.34	0.69	0.38	0.37	0.36	0.40	0.39	0.36	0.35	0.39	0.26	0.32	0.34	0.41	0.33	0.36	
Niacin Equivalent (mgNE)																										
Mean	−	−	−	−	−	−	15.4	15.9	14.7	15.0	16.5	15.1	15.0	14.3	14.5	14.3	14.3	14.4	14.3	13.7	14.2	14.6	14.0	16.2	16.9	0.178
SD	−	−	−	−	−	−	6.1	6.0	5.3	4.9	6.8	5.4	6.1	6.2	5.7	5.7	6.1	5.2	4.9	4.9	4.9	5.4	5.8	5.8	6.3	
Vitamin B6 (mg)																										
Mean	−	−	−	−	−	−	0.70	0.73	0.71	0.71	0.84	0.70	0.67	0.66	0.72	0.71	0.66	0.67	0.67	0.63	0.67	0.69	0.64	0.67	0.69	0.013
SD	−	−	−	−	−	−	0.30	0.30	0.46	0.27	0.71	0.27	0.26	0.33	0.51	0.51	0.28	0.31	0.27	0.25	0.26	0.27	0.28	0.26	0.28	
Vitamin B12 (µg)																										
Mean	−	−	−	−	−	−	4.1	4.0	3.9	3.9	4.6	3.6	3.4	3.7	3.5	3.0	3.1	2.9	2.9	2.9	2.7	2.9	3.0	2.6	2.7	<0.001
SD	−	−	−	−	−	−	3.6	3.7	4.1	3.3	4.7	3.9	3.5	4.2	4.6	3.3	3.1	2.5	2.6	3.2	2.4	2.5	3.0	2.0	2.9	
Folate (µg)																										
Mean	−	−	−	−	−	−	170	172	167	168	177	171	158	157	155	154	149	155	148	151	153	151	149	149	148	<0.001
SD	−	−	−	−	−	−	81	82	72	69	90	73	69	71	64	68	65	60	64	61	69	61	82	62	67	
Pantothenic acid (mg)																										
Mean	−	−	−	−	−	−	4.19	4.26	4.04	4.25	4.23	3.99	3.82	3.64	3.76	3.81	3.80	3.85	3.94	3.64	3.80	3.84	3.63	3.79	3.83	0.006
SD	−	−	−	−	−	−	1.61	1.55	1.34	1.34	1.48	1.37	1.41	1.43	1.28	1.39	1.39	1.35	1.45	1.12	1.23	1.31	1.33	1.28	1.36	
Vitamin C (mg)																										
Mean	82	91	96	89	80	87	61	63	64	60	64	61	55	52	63	52	57	56	49	56	52	52	46	52	49	<0.001
SD	81	103	149	97	83	101	45	42	54	43	48	71	47	37	80	41	80	39	35	42	36	34	34	34	32	
Vitamin D (µg)																										
Mean	−	−	−	−	−	−	4.4	4.4	3.5	4.1	4.6	4.4	3.5	4.0	4.2	3.8	4.0	4.2	3.9	3.1	4.2	3.6	3.7	3.9	3.4	0.03
SD	−	−	−	−	−	−	4.2	6.0	3.6	4.1	5.7	5.5	3.6	4.5	5.5	4.3	4.8	5.1	4.4	2.9	5.3	3.7	4.2	5.2	3.7	
Vitamin E (alpha-tocopherol) (mg)																										
Mean	−	−	−	−	−	−	5.5	5.5	5.3	5.7	4.6	4.7	4.5	4.2	4.2	4.5	4.4	4.2	4.0	4.0	4.0	4.1	3.9	3.7	3.8	<0.001
SD	−	−	−	−	−	−	2.6	2.4	2.3	2.5	2.0	2.1	2.2	2.2	1.9	1.9	2.3	1.9	1.9	1.8	1.7	1.9	2.0	1.7	1.8	
Vitamin K (µg)																										
Mean	−	−	−	−	−	−	142	146	140	134	136	142	115	117	120	118	114	125	113	122	129	130	112	132	128	0.03
SD	−	−	−	−	−	−	131	137	115	116	103	112	82	106	95	84	84	96	81	93	98	97	93	101	105	
Potassium (mg)																										
Mean	−	−	−	−	−	−	1602	1626	1538	1579	1609	1556	1455	1388	1457	1447	1421	1450	1442	1327	1418	1441	1326	1418	1435	0.001
SD	−	−	−	−	−	−	626	613	545	511	565	524	529	528	551	551	508	547	554	448	483	491	554	508	519	
Calcium (mg)																										
Mean	505	515	512	500	493	485	488	481	465	468	461	452	421	396	414	433	422	404	413	387	401	398	369	381	391	<0.001
SD	267	240	255	213	221	238	218	206	224	202	214	233	210	186	199	230	189	190	217	167	179	184	200	199	205	
Magnesium (mg)																										
Mean	−	−	−	−	−	−	161	161	154	154	161	156	144	142	142	145	141	141	140	136	140	146	133	149	143	0.004
SD	−	−	−	−	−	−	61	56	54	49	56	56	50	56	54	59	48	48	52	47	44	47	51	54	49	
Phosphorus (mg)																										
Mean	−	−	−	−	−	−	748	741	701	716	734	699	683	643	654	669	672	658	659	624	649	657	622	635	650	<0.001
SD	−	−	−	−	−	−	273	254	243	229	250	235	251	240	236	246	247	231	241	195	214	217	237	224	230	
Iron (mg)																										
Mean	7.1	7.4	7.3	7.1	7.0	6.9	4.7	4.9	4.6	4.7	4.9	4.8	4.5	4.5	4.5	4.4	4.4	4.1	4.1	4.2	4.1	4.3	4.1	4.1	4.0	<0.001
SD	2.9	3.1	2.9	2.6	2.5	2.8	2.0	1.9	1.7	1.7	2.0	2.0	1.8	2.4	2.0	2.0	1.8	1.6	1.7	1.6	1.4	1.6	1.9	1.5	1.5	
Zinc (mg)																										
Mean	−	−	−	−	−	−	5.5	5.6	5.3	5.5	5.7	5.4	5.4	5.0	5.0	5.1	5.2	5.1	5.2	5.0	5.2	5.3	5.1	5.2	5.2	0.012
SD	−	−	−	−	−	−	2.0	2.0	1.8	1.7	1.9	1.8	2.0	1.8	1.7	1.7	1.8	1.6	1.7	1.7	1.6	1.7	2.0	1.6	1.7	
Copper (mg)																										
Mean	−	−	−	−	−	−	0.73	0.75	0.70	0.72	0.75	0.74	0.67	0.66	0.66	0.66	0.66	0.66	0.65	0.65	0.65	0.69	0.65	0.66	0.66	0.001
SD	−	−	−	−	−	−	0.27	0.28	0.24	0.22	0.26	0.33	0.23	0.24	0.22	0.24	0.22	0.22	0.22	0.21	0.18	0.22	0.22	0.21	0.20	

* Prior to 2000, International Unit (IU) was used as the unit of vitamin A intake. The intakes of niacin equivalent, vitamin B6, vitamin B12, folate, pantothenic acid, vitamin D, vitamin E (alpha-tocopherol), vitamin K, potassium, magnesium, phosphorus, zinc, and copper have been reported since 2001.

**Table 9:** Trends in vitamin and mineral intakes in boys aged 7–14 years in 1995–2019.

Variables	1995	1996	1997	1998	1999	2000	2001	2002	2003	2004	2005	2006	2007	2008	2009	2010	2011	2012	2013	2014	2015	2016	2017	2018	2019	*p* Trend
*n*	803	690	647	705	597	602	591	452	472	419	365	422	392	367	377	390	352	1271	314	320	315	1045	267	273	250	
Vitamin A (retinol equivalent) (IU/μgRAE) *																										
Mean	2936	2654	2908	2703	2610	2521	1051	1109	1004	987	860	668	618	642	581	550	558	585	542	533	598	601	542	521	532	<0.001
SD	2690	2287	3137	2515	1403	1531	675	895	597	600	1583	681	413	828	335	347	334	309	280	327	443	780	275	349	363	
Thiamin (mg)																										
Mean	1.38	1.26	1.28	1.24	1.26	1.27	1.09	1.10	1.12	1.12	1.02	1.27	1.28	0.99	0.98	1.49	1.02	0.97	0.93	0.93	0.95	0.95	0.98	0.97	1.06	<0.001
SD	1.08	0.58	0.51	0.49	0.49	0.71	0.42	0.40	0.80	0.71	0.42	2.32	2.47	0.39	0.40	9.37	0.50	0.40	0.33	0.38	0.40	0.36	0.40	0.41	0.50	
Riboflavin (mg)																										
Mean	1.76	1.65	1.66	1.62	1.64	1.63	1.48	1.48	1.60	1.52	1.44	1.43	1.38	1.39	1.31	1.78	1.32	1.39	1.29	1.28	1.34	1.32	1.38	1.25	1.30	<0.001
SD	1.11	0.57	0.59	0.53	0.56	0.66	0.56	0.52	1.75	1.20	0.58	0.57	0.58	0.58	0.51	8.88	0.48	0.52	0.46	0.46	0.47	0.47	0.55	0.40	0.42	
Niacin Equivalent (mgNE)																										
Mean	−	−	−	−	−	−	26.1	26.0	25.6	25.5	26.2	25.5	25.5	25.1	24.9	25.2	23.8	25.8	25.3	25.1	24.9	25.6	26.0	28.7	29.8	0.941
SD	−	−	−	−	−	−	9.5	8.7	9.6	9.3	8.4	8.8	8.9	7.7	7.9	9.3	7.8	8.1	8.5	9.1	9.1	8.3	9.1	10.7	10.1	
Vitamin B6 (mg)																										
Mean	−	−	−	−	−	−	1.19	1.17	1.59	1.23	1.19	1.14	1.12	1.21	1.14	1.18	1.05	1.13	1.08	1.05	1.08	1.10	1.12	1.08	1.12	0.002
SD	−	−	−	−	−	−	0.45	0.41	5.35	1.04	0.43	0.51	0.53	0.83	0.68	1.30	0.42	0.52	0.36	0.39	0.42	0.38	0.41	0.37	0.42	
Vitamin B12 (µg)																										
Mean	−	−	−	−	−	−	6.7	6.4	6.4	6.0	6.9	6.3	6.0	6.0	6.0	5.5	5.1	5.5	5.2	5.3	5.2	5.6	5.0	5.0	5.9	<0.001
SD	−	−	−	−	−	−	6.5	6.0	5.8	4.4	7.6	5.5	5.5	5.6	5.2	5.1	3.8	4.8	4.3	4.9	4.5	5.1	3.8	3.8	7.3	
Folate (µg)																										
Mean	−	−	−	−	−	−	280	277	273	261	287	253	246	251	238	231	228	248	232	237	245	236	243	236	237	0.002
SD	−	−	−	−	−	−	121	121	113	93	185	115	92	119	86	84	80	89	78	80	96	108	89	94	86	
Pantothenic acid (mg)																										
Mean	−	−	−	−	−	−	6.93	6.82	6.64	6.62	6.79	6.55	6.39	6.34	6.09	6.13	6.04	6.56	6.42	6.22	6.36	6.42	6.55	6.26	6.40	0.02
SD	−	−	−	−	−	−	2.42	2.10	2.11	1.97	2.20	1.82	1.93	1.75	1.65	1.71	1.64	1.83	1.82	1.74	2.02	1.85	2.11	1.82	1.87	
Vitamin C (mg)																										
Mean	140	134	136	112	123	119	85	84	89	89	95	77	83	84	88	80	83	75	68	65	67	66	71	67	69	<0.001
SD	123	152	140	95	102	105	47	58	82	99	140	63	104	86	114	118	179	49	42	45	45	47	44	38	47	
Vitamin D (µg)																										
Mean	−	−	−	−	−	−	6.0	5.8	6.1	5.4	6.1	6.1	5.7	6.7	6.0	6.4	5.5	6.6	6.2	5.5	6.0	7.1	6.0	5.4	5.6	0.74
SD	−	−	−	−	−	−	6.3	6.3	7.6	6.1	7.1	6.9	6.7	6.4	5.6	6.7	4.7	7.1	6.8	6.0	5.8	7.8	6.5	7.3	6.0	
Vitamin E (alpha-tocopherol) (mg)																										
Mean	−	−	−	−	−	−	8.7	8.5	8.6	9.1	7.0	6.9	6.7	6.8	6.5	6.6	6.3	6.6	6.1	6.3	6.5	6.1	6.6	5.9	6.0	<0.001
SD	−	−	−	−	−	−	3.7	3.5	5.6	7.9	3.0	3.2	3.1	3.5	2.9	3.6	2.7	2.8	2.5	2.4	2.7	2.6	3.0	2.6	2.5	
Vitamin K (µg)																										
Mean	−	−	−	−	−	−	231	213	210	193	189	197	195	179	180	183	180	200	187	202	202	196	208	189	196	0.489
SD	−	−	−	−	−	−	193	160	176	143	115	146	137	117	118	128	127	140	126	123	145	128	148	135	127	
Potassium (mg)																										
Mean	−	−	−	−	−	−	2636	2545	2490	2472	2496	2396	2356	2309	2266	2221	2165	2335	2259	2188	2297	2257	2338	2267	2307	0.004
SD	−	−	−	−	−	−	911	825	792	760	744	699	756	665	701	683	628	670	620	636	778	650	698	662	653	
Calcium (mg)																										
Mean	733	705	705	710	706	686	786	770	744	764	760	752	711	693	670	673	677	704	667	640	689	678	698	668	676	0.002
SD	311	258	287	269	276	257	314	305	278	308	306	286	286	252	264	267	251	258	246	230	254	245	265	254	238	
Magnesium (mg)																										
Mean	−	−	−	−	−	−	270	262	255	255	257	243	238	237	228	226	220	239	230	227	236	232	241	250	236	0.017
SD	−	−	−	−	−	−	90	83	82	76	71	75	75	65	64	68	63	69	65	65	78	64	76	93	64	
Phosphorus (mg)																										
Mean	−	−	−	−	−	−	1242	1217	1176	1183	1195	1180	1140	1127	1095	1104	1081	1156	1120	1097	1125	1129	1147	1106	1128	0.01
SD	−	−	−	−	−	−	422	367	356	352	344	328	327	292	294	310	294	305	318	323	327	303	340	329	314	
Iron (mg)																										
Mean	11.5	10.7	10.8	10.4	10.5	10.7	7.7	7.5	11.3	7.2	7.7	7.3	7.3	7.4	7.0	7.0	6.8	7.0	6.9	6.8	6.9	6.9	7.0	6.5	6.7	<0.001
SD	6.0	3.6	3.5	3.3	3.1	4.1	2.9	2.8	74.1	2.4	2.9	2.7	2.7	2.5	2.4	2.6	2.6	2.3	2.2	2.3	2.4	2.4	2.4	2.1	2.0	
Zinc (mg)																										
Mean	−	−	−	−	−	−	9.7	9.7	9.5	9.4	9.8	9.2	9.3	9.0	8.9	8.9	8.9	9.3	9.2	9.3	9.2	9.2	9.5	9.1	9.3	0.079
SD	−	−	−	−	−	−	3.3	3.2	3.2	2.8	3.2	2.5	3.0	2.8	2.7	2.6	2.6	2.7	3.0	3.1	2.9	2.6	3.3	3.0	3.0	
Copper (mg)																										
Mean	−	−	−	−	−	−	1.28	1.25	1.20	1.21	1.23	1.13	1.13	1.14	1.09	1.10	1.07	1.15	1.12	1.12	1.12	1.13	1.16	1.10	1.11	0.008
SD	−	−	−	−	−	−	0.48	0.42	0.41	0.37	0.35	0.35	0.35	0.33	0.31	0.32	0.33	0.35	0.33	0.31	0.37	0.35	0.37	0.34	0.33	

* Prior to 2000, International Unit (IU) was used as the unit of vitamin A intake. The intakes of niacin equivalent, vitamin B6, vitamin B12, folate, pantothenic acid, vitamin D, vitamin E (alpha-tocopherol), vitamin K, potassium, magnesium, phosphorus, zinc, and copper have been reported since 2001.

**Table 10:** Trends in vitamin and mineral intakes in girls aged 7–14 years in 1995–2019.

Variables	1995	1996	1997	1998	1999	2000	2001	2002	2003	2004	2005	2006	2007	2008	2009	2010	2011	2012	2013	2014	2015	2016	2017	2018	2019	*p* Trend
*n*	739	668	628	675	535	581	580	464	467	351	376	393	403	337	382	349	368	1285	295	300	282	943	245	244	204	
Vitamin A (retinol equivalent) (IU/μgRAE) *																										
Mean	2659	2586	2670	2562	2498	2450	970	980	945	908	709	604	606	532	582	571	548	532	534	514	524	519	512	479	491	<0.001
SD	1963	1872	3160	2755	1660	1366	542	541	507	457	605	561	546	427	440	667	321	295	332	278	368	639	293	260	255	
Thiamin (mg)																										
Mean	1.22	1.19	1.15	1.14	1.16	1.14	1.01	0.96	0.95	0.98	0.90	1.19	1.19	0.85	1.05	0.89	0.96	0.85	0.85	0.86	0.84	0.87	0.86	0.88	0.94	<0.001
SD	0.64	0.54	0.41	0.44	0.52	0.51	0.42	0.34	0.41	0.36	0.57	2.50	2.53	0.31	1.50	0.33	0.55	0.29	0.29	0.29	0.31	0.29	0.31	0.32	0.40	
Riboflavin (mg)																										
Mean	1.57	1.55	1.52	1.50	1.49	1.45	1.32	1.31	1.27	1.33	1.29	1.29	1.33	1.22	1.29	1.22	1.25	1.21	1.13	1.19	1.18	1.18	1.24	1.12	1.18	<0.001
SD	0.61	0.53	0.52	0.53	0.53	0.53	0.49	0.45	0.45	0.58	0.95	0.61	1.59	0.52	1.09	0.57	0.51	0.46	0.35	0.41	0.43	0.43	0.39	0.34	0.35	
Niacin Equivalent (mgNE)																										
Mean	−	−	−	−	−	−	23.3	23.1	23.3	23.4	23.6	24.1	22.8	22.0	23.5	22.4	22.5	22.5	22.5	22.3	21.8	22.2	22.9	25.5	27.4	0.456
SD	−	−	−	−	−	−	7.3	7.3	8.2	7.5	8.0	8.5	7.1	7.1	8.3	7.8	7.1	6.2	7.0	6.8	7.2	6.5	8.2	8.0	7.9	
Vitamin B6 (mg)																										
Mean	−	−	−	−	−	−	1.06	1.05	1.07	1.15	1.23	1.08	1.12	1.02	1.09	1.07	1.04	1.04	0.97	0.97	0.96	0.98	0.99	0.97	1.03	0.002
SD	−	−	−	−	−	−	0.36	0.35	0.46	1.03	2.63	0.64	2.14	0.68	0.80	0.93	0.61	0.64	0.33	0.29	0.35	0.33	0.34	0.33	0.32	
Vitamin B12 (µg)																										
Mean	−	−	−	−	−	−	5.7	5.6	6.1	5.4	6.1	6.3	5.6	5.0	5.4	5.1	5.2	4.7	4.8	4.9	4.8	4.8	5.4	4.9	5.8	0.021
SD	−	−	−	−	−	−	4.7	4.4	5.2	4.5	5.7	7.0	5.3	4.3	4.7	4.7	4.6	4.2	4.1	4.4	4.4	4.8	5.2	5.3	8.0	
Folate (µg)																										
Mean	−	−	−	−	−	−	259	255	255	245	265	250	236	227	237	228	224	229	222	228	226	218	226	218	230	0.001
SD	−	−	−	−	−	−	97	98	94	76	108	127	97	95	88	103	77	78	77	70	90	93	86	74	76	
Pantothenic acid (mg)																										
Mean	−	−	−	−	−	−	5.99	6.06	5.82	5.96	5.99	5.97	5.80	5.60	5.72	5.62	5.59	5.70	5.60	5.63	5.61	5.59	5.81	5.57	5.83	0.007
SD	−	−	−	−	−	−	1.83	1.91	1.76	1.58	1.62	1.70	1.68	1.64	1.56	1.64	1.48	1.50	1.45	1.34	1.70	1.47	1.52	1.49	1.55	
Vitamin C (mg)																										
Mean	132	126	122	119	119	114	85	81	90	87	99	80	79	79	106	78	76	74	69	72	69	68	69	64	66	<0.001
SD	133	95	89	109	117	82	60	50	84	82	146	67	85	81	199	103	90	62	46	45	53	38	48	41	35	
Vitamin D (µg)																										
Mean	−	−	−	−	−	−	5.7	5.6	6.1	5.5	5.4	6.6	4.8	6.3	6.2	5.5	5.9	5.6	5.5	5.5	5.5	5.9	5.4	5.2	5.8	0.305
SD	−	−	−	−	−	−	6.0	5.9	6.1	6.3	6.7	7.9	5.9	6.3	6.8	5.6	5.9	5.9	5.9	5.8	6.3	6.4	5.2	5.6	6.7	
Vitamin E (alpha-tocopherol) (mg)																										
Mean	−	−	−	−	−	−	8.1	7.9	7.9	7.7	6.5	6.6	6.3	6.2	6.9	5.7	6.2	6.2	5.7	5.8	5.8	5.9	6.1	5.5	5.9	<0.001
SD	−	−	−	−	−	−	3.2	3.0	3.3	2.9	2.6	2.7	2.9	3.8	15.0	2.5	2.5	2.6	2.4	2.2	2.5	2.5	2.9	2.2	2.3	
Vitamin K (µg)																										
Mean	−	−	−	−	−	−	202	190	213	183	189	188	174	164	173	165	174	183	175	176	175	162	166	172	204	0.05
SD	−	−	−	−	−	−	144	151	164	124	120	117	112	117	108	106	114	117	128	112	120	99	108	115	141	
Potassium (mg)																										
Mean	−	−	−	−	−	−	2347	2300	2261	2283	2314	2224	2145	2056	2146	2056	2077	2106	2047	2078	2057	2050	2100	2046	2133	0.001
SD	−	−	−	−	−	−	750	736	716	655	701	631	701	715	622	612	597	620	566	539	688	594	617	583	565	
Calcium (mg)																										
Mean	674	665	671	640	639	625	686	671	650	688	665	669	623	614	625	617	641	620	607	609	620	610	646	603	594	<0.001
SD	265	252	246	245	241	229	267	256	268	251	223	245	232	233	245	222	230	207	206	197	226	214	206	219	198	
Magnesium (mg)																										
Mean	−	−	−	−	−	−	241	235	232	236	236	224	216	210	216	207	211	215	211	212	208	209	214	223	214	0.006
SD	−	−	−	−	−	−	74	72	71	63	65	63	66	70	61	59	60	62	61	50	67	58	64	70	57	
Phosphorus (mg)																										
Mean	−	−	−	−	−	−	1089	1073	1045	1079	1058	1078	1033	995	1034	1001	1023	1012	1007	1007	1002	996	1023	987	1014	0.002
SD	−	−	−	−	−	−	338	312	317	289	277	288	282	281	271	261	272	244	256	234	291	246	249	259	245	
Iron (mg)																										
Mean	10.3	10.2	10.1	9.9	9.8	9.8	7.0	6.9	6.9	6.7	6.9	7.0	6.6	6.5	6.9	6.4	6.7	6.3	6.3	6.3	6.3	6.1	6.5	6.1	6.3	<0.001
SD	3.5	3.4	3.1	3.1	3.2	3.4	2.3	2.3	2.6	2.0	2.2	2.4	2.1	2.4	2.4	2.3	2.5	2.0	2.2	1.8	2.3	1.9	2.3	2.0	2.0	
Zinc (mg)																										
Mean	−	−	−	−	−	−	8.4	8.5	8.4	8.4	8.5	8.4	8.2	7.8	8.2	7.9	8.1	8.1	8.0	8.0	8.0	7.9	8.3	8.0	8.3	0.013
SD	−	−	−	−	−	−	2.5	2.5	2.9	2.4	2.3	2.4	2.2	2.3	2.2	2.1	2.1	2.1	2.1	1.9	2.4	1.9	2.2	2.2	2.4	
Copper (mg)																										
Mean	−	−	−	−	−	−	1.12	1.12	1.07	1.10	1.10	1.06	1.03	1.01	1.02	0.99	1.00	1.01	0.99	1.00	1.01	0.99	1.03	0.98	1.00	0.001
SD	−	−	−	−	−	−	0.36	0.35	0.34	0.31	0.31	0.47	0.32	0.33	0.30	0.27	0.29	0.29	0.29	0.25	0.32	0.27	0.30	0.28	0.30	

* Prior to 2000, International Unit (IU) was used as the unit of vitamin A intake. The intakes of niacin equivalent, vitamin B6, vitamin B12, folate, pantothenic acid, vitamin D, vitamin E (alpha-tocopherol), vitamin K, potassium, magnesium, phosphorus, zinc, and copper have been reported since 2001.

**Table 11:** Trends in vitamin and mineral intakes in adolescent boys aged 15–19 years in 1995–2019.

Variables	1995	1996	1997	1998	1999	2000	2001	2002	2003	2004	2005	2006	2007	2008	2009	2010	2011	2012	2013	2014	2015	2016	2017	2018	2019	*p* Trend
*n*	500	473	446	416	373	339	358	301	272	239	218	239	201	190	206	193	193	702	175	173	165	559	141	143	130	
Vitamin A (retinol equivalent) (IU/μgRAE) *																										
Mean	3116	3088	3187	2711	3367	2725	978	927	980	938	630	576	805	724	550	563	568	597	563	535	605	542	574	539	529	<0.001
SD	4269	2999	4098	2206	6364	2134	1008	719	1022	1129	700	422	1926	1729	546	753	426	534	680	622	390	512	668	534	752	
Thiamin (mg)																										
Mean	1.55	1.53	1.56	1.46	1.50	1.45	1.15	1.13	1.22	1.16	1.18	1.41	1.42	1.11	1.20	1.59	1.18	1.15	1.08	1.03	1.25	1.12	1.14	1.26	1.17	0.007
SD	0.68	0.71	0.76	0.68	0.76	0.73	0.55	0.51	1.04	0.70	0.58	1.91	2.24	0.58	0.93	6.20	1.56	0.60	0.52	0.43	0.58	0.57	0.55	0.62	0.59	
Riboflavin (mg)																										
Mean	1.76	1.74	1.76	1.66	1.80	1.72	1.45	1.44	1.57	1.70	1.42	1.53	1.75	1.32	1.43	1.42	1.50	1.37	1.25	1.26	1.42	1.24	1.34	1.34	1.32	<0.001
SD	0.76	0.74	0.80	0.70	0.93	0.84	0.68	0.62	0.95	2.71	0.74	1.49	3.06	0.60	0.88	1.17	2.23	0.66	0.59	0.55	0.61	0.51	0.62	0.59	0.58	
Niacin Equivalent (mgNE)																										
Mean	−	−	−	−	−	−	31.0	30.8	31.7	29.7	30.4	30.4	31.1	28.8	29.9	29.5	28.7	30.0	30.1	28.2	33.0	30.0	31.4	37.6	36.7	0.487
SD	−	−	−	−	−	−	11.6	11.0	11.7	10.3	10.7	11.2	11.3	9.4	12.3	11.4	9.8	9.9	10.8	9.6	11.4	11.0	10.9	16.0	15.2	
Vitamin B6 (mg)																										
Mean	−	−	−	−	−	−	1.29	1.25	1.36	1.46	1.29	1.64	1.68	1.23	1.36	1.41	1.45	1.29	1.21	1.11	1.33	1.23	1.27	1.36	1.31	0.296
SD	−	−	−	−	−	−	0.54	0.47	0.87	1.88	0.75	2.34	3.34	0.54	1.04	1.63	3.06	0.80	0.48	0.43	0.50	0.45	0.50	0.60	0.63	
Vitamin B12 (µg)																										
Mean	−	−	−	−	−	−	8.1	7.6	8.4	6.6	6.9	6.6	7.5	6.4	6.2	5.2	5.1	5.9	5.5	5.4	5.5	5.5	6.2	5.6	4.9	0.001
SD	−	−	−	−	−	−	9.8	6.5	8.5	6.5	6.6	5.3	9.7	8.4	8.1	5.8	4.1	5.7	5.0	4.8	4.5	5.0	6.3	4.7	3.9	
Folate (µg)																										
Mean	−	−	−	−	−	−	303	286	303	282	279	277	290	274	279	261	257	273	261	257	296	252	275	276	260	0.006
SD	−	−	−	−	−	−	175	128	161	154	142	119	215	207	131	145	111	116	110	120	123	111	129	117	114	
Pantothenic acid (mg)																										
Mean	−	−	−	−	−	−	7.07	6.92	7.16	6.72	6.60	6.70	6.78	6.31	6.59	6.63	6.39	6.70	6.43	6.26	7.21	6.47	6.81	6.93	6.85	0.572
SD	−	−	−	−	−	−	2.87	2.51	2.78	2.41	2.47	2.41	2.60	2.41	2.35	2.61	2.23	2.18	2.22	2.34	2.44	2.18	2.42	2.55	2.49	
Vitamin C (mg)																										
Mean	164	148	145	139	148	136	89	85	88	112	100	112	108	88	110	121	86	86	72	68	89	71	80	80	75	<0.001
SD	178	150	188	158	221	162	70	61	68	150	137	214	216	126	188	246	85	68	45	47	63	46	55	47	54	
Vitamin D (µg)																										
Mean	−	−	−	−	−	−	8.4	7.8	8.2	7.3	7.3	7.9	6.6	6.6	7.1	6.1	6.4	6.8	6.9	6.4	6.9	7.3	7.2	6.9	5.9	0.011
SD	−	−	−	−	−	−	9.3	7.7	8.5	8.6	8.1	10.0	7.0	7.8	9.8	7.3	7.4	7.9	7.9	7.1	7.5	8.1	7.7	8.6	7.1	
Vitamin E (alpha-tocopherol) (mg)																										
Mean	−	−	−	−	−	−	10.1	9.6	10.1	9.7	8.2	9.3	9.3	7.6	8.0	7.9	7.2	7.8	7.0	7.0	8.2	6.9	7.3	7.2	7.3	0.001
SD	−	−	−	−	−	−	4.7	4.3	4.4	4.3	3.7	12.9	22.5	4.2	3.8	4.7	3.0	3.6	3.3	3.5	3.6	3.0	3.4	3.5	3.3	
Vitamin K (µg)																										
Mean	−	−	−	−	−	−	254	236	240	206	233	240	229	205	212	204	201	215	196	217	269	210	245	228	237	0.648
SD	−	−	−	−	−	−	179	187	190	144	190	164	165	139	149	140	139	134	128	146	183	130	189	141	180	
Potassium (mg)																										
Mean	−	−	−	−	−	−	2530	2460	2559	2376	2387	2383	2329	2223	2367	2257	2244	2328	2225	2136	2506	2219	2301	2437	2280	0.032
SD	−	−	−	−	−	−	1054	939	1037	891	928	898	848	908	972	947	879	841	805	844	922	788	884	991	894	
Calcium (mg)																										
Mean	631	639	640	613	666	616	633	621	642	620	597	592	578	517	572	531	565	550	502	536	578	508	528	523	504	<0.001
SD	349	330	348	330	387	369	364	372	400	334	351	344	375	257	323	300	319	276	256	378	350	264	300	276	274	
Magnesium (mg)																										
Mean	−	−	−	−	−	−	275	265	269	253	253	259	248	239	252	245	241	250	240	237	266	242	244	278	239	0.051
SD	−	−	−	−	−	−	103	101	105	82	90	95	82	82	94	95	86	81	81	100	105	80	85	117	85	
Phosphorus (mg)																										
Mean	−	−	−	−	−	−	1279	1242	1274	1204	1190	1199	1192	1117	1187	1162	1148	1173	1140	1116	1258	1144	1184	1202	1181	0.035
SD	−	−	−	−	−	−	508	453	463	408	416	415	409	356	427	451	390	361	379	401	427	366	410	444	414	
Iron (mg)																										
Mean	13.3	12.8	13.1	12.3	12.5	12.7	8.6	8.5	9.4	8.0	8.2	8.5	8.4	7.9	8.1	7.9	7.8	8.2	7.8	8.0	8.6	7.8	8.1	8.3	7.9	<0.001
SD	5.1	4.6	5.0	4.2	5.0	4.9	3.5	3.6	13.1	2.7	3.1	3.2	3.2	3.1	3.3	2.9	2.9	3.1	2.8	4.4	3.1	2.6	2.7	3.4	2.9	
Zinc (mg)																										
Mean	−	−	−	−	−	−	11.2	11.1	11.4	10.5	10.8	11.2	11.0	10.4	10.8	10.9	10.5	10.8	10.7	10.6	11.6	10.8	10.9	11.6	11.4	0.789
SD	−	−	−	−	−	−	4.5	3.9	4.2	3.6	3.9	3.6	4.2	3.4	4.0	3.8	3.4	3.5	3.6	4.1	4.0	3.2	3.4	4.5	4.1	
Copper (mg)																										
Mean	−	−	−	−	−	−	1.47	1.40	1.40	1.32	1.36	1.38	1.32	1.30	1.36	1.36	1.30	1.33	1.35	1.27	1.41	1.30	1.34	1.34	1.29	0.03
SD	−	−	−	−	−	−	0.70	0.49	0.48	0.42	0.48	0.44	0.44	0.40	0.51	0.54	0.40	0.41	0.49	0.50	0.46	0.40	0.47	0.45	0.42	

* Prior to 2000, International Unit (IU) was used as the unit of vitamin A intake. The intakes of niacin equivalent, vitamin B6, vitamin B12, folate, pantothenic acid, vitamin D, vitamin E (alpha-tocopherol), vitamin K, potassium, magnesium, phosphorus, zinc, and copper have been reported since 2001.

**Table 12:** Trends in vitamin and mineral intakes in adolescent girls aged 15–19 years in 1995–2019.

Variables	1995	1996	1997	1998	1999	2000	2001	2002	2003	2004	2005	2006	2007	2008	2009	2010	2011	2012	2013	2014	2015	2016	2017	2018	2019	*p* Trend
*n*	440	438	418	411	372	369	330	314	291	196	211	219	192	170	197	193	187	599	162	182	169	491	142	134	119	
Vitamin A (retinol equivalent) (IU/μgRAE) *																										
Mean	2807	2547	2334	2338	2482	2245	875	843	857	840	538	538	561	691	476	494	467	491	451	455	416	450	487	420	446	<0.001
SD	2712	2895	1790	1799	3742	1726	644	676	789	993	419	453	498	1643	388	665	344	363	317	338	238	350	428	255	292	
Thiamin (mg)																										
Mean	1.23	1.18	1.15	1.11	1.19	1.15	0.91	0.84	1.11	1.40	0.91	0.88	0.95	0.81	0.86	1.02	0.96	0.87	0.83	0.76	0.83	0.83	0.85	0.91	0.98	0.001
SD	0.80	0.60	0.51	0.45	0.58	0.86	0.39	0.34	2.72	4.10	0.51	0.42	0.67	0.33	0.52	1.63	0.83	0.47	0.39	0.28	0.32	0.32	0.37	0.36	0.41	
Riboflavin (mg)																										
Mean	1.48	1.35	1.31	1.33	1.37	1.35	1.18	1.13	1.56	1.56	1.20	1.17	1.27	1.11	1.24	1.22	1.23	1.10	1.05	1.06	1.07	1.02	1.15	1.02	1.11	<0.001
SD	0.83	0.54	0.53	0.53	0.62	0.87	0.49	0.46	3.64	3.19	0.66	0.60	0.76	0.51	2.24	1.28	1.14	0.51	0.45	0.44	0.44	0.40	0.52	0.38	0.40	
Niacin Equivalent (mgNE)																										
Mean	−	−	−	−	−	−	25.0	23.9	24.3	25.8	25.4	24.3	24.7	22.8	23.2	22.7	22.9	23.4	23.3	21.8	24.1	23.1	23.1	27.0	30.1	0.466
SD	−	−	−	−	−	−	9.8	8.4	8.6	9.0	9.1	8.5	9.3	7.6	7.8	8.3	8.3	7.9	8.3	7.4	8.0	8.1	7.5	9.2	11.1	
Vitamin B6 (mg)																										
Mean	−	−	−	−	−	−	1.07	0.99	1.28	1.46	1.17	1.08	1.16	1.03	1.75	1.22	1.06	1.03	1.00	0.93	1.00	0.95	0.97	1.00	1.09	0.036
SD	−	−	−	−	−	−	0.44	0.40	2.66	3.17	0.79	0.94	0.91	0.54	7.74	2.22	0.91	0.54	0.38	0.34	0.36	0.37	0.37	0.37	0.41	
Vitamin B12 (µg)																										
Mean	−	−	−	−	−	−	6.4	6.2	6.3	5.8	5.8	5.5	5.2	5.8	4.9	4.7	4.1	4.7	4.2	4.7	4.7	4.2	3.7	3.9	4.4	<0.001
SD	−	−	−	−	−	−	6.4	6.6	6.4	5.0	5.8	5.9	5.0	7.5	4.7	5.6	3.5	4.8	3.9	5.2	4.1	4.1	2.8	3.9	4.0	
Folate (µg)																										
Mean	−	−	−	−	−	−	268	249	264	258	271	270	249	259	233	237	236	239	229	237	234	228	235	237	245	0.003
SD	−	−	−	−	−	−	135	118	144	125	111	133	98	177	90	126	98	96	98	101	89	97	112	99	93	
Pantothenic acid (mg)																										
Mean	−	−	−	−	−	−	5.60	5.32	5.40	5.56	5.52	5.40	5.34	5.12	5.03	5.05	5.14	5.23	5.10	5.07	5.19	4.97	5.30	5.19	5.60	0.074
SD	−	−	−	−	−	−	2.04	1.91	1.95	1.95	1.72	1.71	1.78	1.66	1.57	1.77	1.69	1.74	1.70	1.63	1.68	1.62	1.82	1.62	1.76	
Vitamin C (mg)																										
Mean	137	121	122	118	123	115	91	78	88	100	100	98	91	85	84	93	79	78	70	74	69	69	68	67	81	<0.001
SD	128	98	110	119	121	121	67	64	83	129	108	111	103	85	89	135	79	55	57	50	42	49	40	42	49	
Vitamin D (µg)																										
Mean	−	−	−	−	−	−	8.1	7.2	7.2	7.1	6.7	6.1	6.1	7.2	5.7	5.3	4.8	6.0	6.0	5.5	6.1	5.7	5.0	4.1	5.3	<0.001
SD	−	−	−	−	−	−	9.0	8.0	7.8	8.4	7.7	7.2	7.7	8.4	7.2	6.6	5.3	7.4	7.1	6.7	6.4	7.6	6.4	5.2	6.3	
Vitamin E (alpha-tocopherol) (mg)																										
Mean	−	−	−	−	−	−	8.7	8.0	8.3	8.6	7.4	7.0	6.9	6.3	6.6	6.5	7.9	6.3	6.1	6.2	6.7	5.9	6.3	6.1	6.6	0.001
SD	−	−	−	−	−	−	3.8	3.4	4.1	3.8	3.4	3.1	3.0	2.8	2.9	3.1	22.2	2.7	2.5	3.0	2.9	2.6	3.3	2.5	3.1	
Vitamin K (µg)																										
Mean	−	−	−	−	−	−	237	206	216	200	212	214	197	175	192	189	204	201	192	188	193	190	191	213	215	0.143
SD	−	−	−	−	−	−	191	167	197	163	137	159	137	113	119	146	130	136	145	141	138	139	139	132	153	
Potassium (mg)																										
Mean	−	−	−	−	−	−	2205	1991	2029	2085	2132	2051	2052	1930	1886	1861	1935	1951	1850	1876	1905	1782	1919	1934	2060	0.019
SD	−	−	−	−	−	−	873	788	799	782	798	771	765	696	682	711	731	719	677	682	634	664	693	808	703	
Calcium (mg)																										
Mean	545	512	488	502	498	477	516	487	518	498	496	503	493	443	447	452	496	456	431	447	434	426	462	424	454	<0.001
SD	282	248	236	235	262	263	280	266	321	265	240	274	257	210	255	246	261	237	207	232	227	215	249	221	210	
Magnesium (mg)																										
Mean	−	−	−	−	−	−	231	213	217	218	221	215	216	206	198	196	203	203	197	200	205	189	201	221	213	0.028
SD	−	−	−	−	−	−	82	81	79	75	75	75	83	67	67	68	69	70	65	69	73	65	73	92	65	
Phosphorus (mg)																										
Mean	−	−	−	−	−	−	1020	967	981	1000	993	974	955	909	906	912	919	927	898	883	927	888	933	903	985	0.014
SD	−	−	−	−	−	−	373	333	319	328	306	318	322	262	297	300	299	300	275	276	296	290	292	272	303	
Iron (mg)																										
Mean	11.2	10.6	10.5	10.3	10.4	10.1	7.4	7.1	7.3	7.2	7.4	7.2	7.2	7.1	7.2	6.7	7.0	6.7	6.8	6.6	7.0	6.5	6.7	6.7	7.0	<0.001
SD	4.9	4.1	3.7	3.4	3.9	4.9	2.8	2.7	2.9	2.7	2.5	2.5	3.1	3.0	7.8	2.4	2.8	2.2	2.5	2.3	2.4	2.3	2.5	2.2	2.2	
Zinc (mg)																										
Mean	−	−	−	−	−	−	8.4	8.1	8.3	8.5	8.8	8.5	8.2	7.8	8.0	8.0	7.9	8.1	8.1	7.6	8.2	7.8	8.3	8.3	8.6	0.346
SD	−	−	−	−	−	−	2.9	2.6	3.0	2.8	3.2	2.7	2.7	2.3	2.7	2.4	2.4	2.6	2.7	2.3	2.5	2.5	2.9	2.4	2.8	
Copper (mg)																										
Mean	−	−	−	−	−	−	1.13	1.06	1.10	1.09	1.10	1.12	1.03	1.02	1.03	1.04	1.03	1.01	1.00	1.02	1.06	0.97	1.03	1.03	1.05	0.012
SD	−	−	−	−	−	−	0.41	0.37	0.53	0.35	0.37	0.50	0.33	0.29	0.32	0.38	0.33	0.31	0.32	0.32	0.30	0.32	0.36	0.27	0.31	

* Prior to 2000, International Unit (IU) was used as the unit of vitamin A intake. The intakes of niacin equivalent, vitamin B6, vitamin B12, folate, pantothenic acid, vitamin D, vitamin E (alpha-tocopherol), vitamin K, potassium, magnesium, phosphorus, zinc, and copper have been reported since 2001.

## Data Availability

The data used in this study can be obtained from the survey reports of the Ministry of Health, Labor and Welfare (https://www.mhlw.go.jp/bunya/kenkou/kenkou_eiyou_chousa.html, accessed on 19 December 2022).
